# The efficacy and safety of Yupingfengsan in the treatment of allergic rhinitis: a systematic review and meta-analysis

**DOI:** 10.3389/fphar.2025.1628640

**Published:** 2025-09-15

**Authors:** Yi Wang, Yumei Tang, Jili Xu, Yangziting Bu, Lan Luo, Jie Wu

**Affiliations:** ^1^ Chengdu University of Traditional Chinese Medicine, Chengdu, Sichuan, China; ^2^ Hospital of Chengdu University of Traditional Chinese Medicine, Chengdu, Sichuan, China

**Keywords:** allergic rhinitis, meta-analysis, systematic review, traditional Chinese medicine, Yupingfengsan

## Abstract

**Background:**

Allergic rhinitis (AR) is a chronic, non-infectious inflammation of the nasal mucosa, primarily mediated by immunoglobulin E (IgE) following allergen exposure in atopic individuals. Yupingfengsan (YPFS), a classical traditional Chinese medicine (TCM) formula, has been used to manage AR. However, its efficacy and safety require comprehensive evaluation.

**Methods:**

This review was registered in PROSPERO (CRD420251009897). Eight databases were systematically searched up to December 2024 for randomized controlled trials (RCTs) evaluating YPFS for AR. Meta-analyses were conducted using Review Manager 5.4 and Stata 18.0. Subgroup and sensitivity analyses explored heterogeneity and result stability. Publication bias was assessed using funnel plots and Egger’s test. Evidence quality was appraised with GRADEpro, and potential mechanisms of YPFS in AR were summarized.

**Results:**

Thirty-nine RCTs involving 4,578 participants met the inclusion criteria. YPFS combined with conventional pharmacotherapy significantly improved Total Nasal Symptom Score (TNSS), regulated Th1/Th2 and Treg/Th17 balance, and reduced serum IgE, IL-4, and IL-6 levels compared with conventional pharmacotherapy alone. As monotherapy, YPFS improved TNSS and lowered IgE, IL-4, and IL-6 levels more effectively than conventional treatment, though its effects on immune balance and IL-4 modulation remain uncertain due to limited data. Both regimens increased overall clinical effectiveness and reduced relapse rates. No specific adverse reactions to YPFS were reported in studies that monitored safety; however, many trials did not report adverse events, limiting conclusions about its safety profile and long-term tolerability.

**Conclusion:**

YPFS, particularly when combined with conventional pharmacotherapy, offers superior benefits over conventional treatment alone in modulating immune function, reducing inflammation, improving clinical outcomes, and lowering relapse risk in AR. YPFS monotherapy also shows potential immunomodulatory and anti-inflammatory effects. Current safety data do not indicate major concerns, but incomplete reporting and limited immune parameter data restrict definitive conclusions. High-quality, rigorously monitored RCTs are needed to confirm these findings and better define the safety of YPFS in clinical use.

**Systematic Evaluation Registry:**

https://www.crd.york.ac.uk/PROSPERO/view/CRD420251009897. Identifier [CRD420251009897].

## 1 Introduction

Allergic rhinitis (AR) is a chronic, non-infectious inflammation of the nasal mucosa triggered by allergens such as pollen and dust mites. It is mediated by immunoglobulin E (IgE) and typically presents with paroxysmal sneezing, watery nasal discharge, nasal congestion, and itching. The condition is influenced by genetic, environmental, and immunological factors (Bousquet et al., 2020). Its pathophysiology involves an IgE-mediated Th2-type inflammatory response with eosinophil infiltration, neurogenic inflammation, and mucosal barrier disruption, ultimately leading to chronic inflammation and tissue remodeling of the nasal mucosa (Dong et al., 2024). AR affects 10%–40% of the global population (Brożek et al., 2017) and is associated with substantial socioeconomic burden. In the European Union, reduced productivity due to AR is estimated to cause annual economic losses between €5.5 and €151 billion (Zuberbier et al., 2014). In the United States, direct medical costs reach approximately $1.15 billion annually (Cox, 2016). Beyond its impact on quality of life, AR is linked to comorbidities such as asthma, eczema, chronic or recurrent sinusitis, cough, and various headache syndromes (Bernstein et al., 2024). Addressing its prevention, control, and complications remains a public health priority worldwide.

Current pharmacological treatments include intranasal or oral corticosteroids, antihistamines, leukotriene receptor antagonists, mast cell stabilizers, decongestants, and anticholinergics. While these agents act rapidly and effectively, they are associated with high recurrence rates and potential adverse effects during long-term use, including nasal bleeding, mucosal atrophy, headache, and drug resistance (Seidman et al., 2015). In children, prolonged intranasal corticosteroid use may impair growth (Lee et al., 2014). These limitations underscore the need for safer and more sustainable therapeutic options.

Traditional Chinese medicine (TCM) offers a complementary approach rooted in centuries of clinical experience. Yupingfengsan (YPFS), first described in Zhu Danxi’s Danxi Xinfa during the Yuan Dynasty, contains Astragali mongholici radix, Atractylodis macrocephalae rhizoma, and Saposhnikoviae radix. It follows the therapeutic principles of “benefiting qi, consolidating the exterior, dispelling wind, and eliminating pathogenic factors” (Wang et al., 2015). Clinically, YPFS has been used to treat conditions characterized by exterior deficiency and has demonstrated benefits in AR by improving symptoms and modulating immune and inflammatory markers (Wu and Bai, 2021; Wang, 2018; Zhang et al., 2018). However, the evidence remains limited by small sample sizes, heterogeneity in study design, variation in outcome measures, and uncertain methodological quality.

This study aims to systematically review and meta-analyze randomized controlled trials (RCTs) assessing the efficacy and safety of YPFS in AR. By quantitatively synthesizing clinical outcomes, summarizing potential mechanisms, and evaluating safety data, we seek to provide a robust evidence base to guide future research and inform clinical practice.

## 2 Materials and methods

### 2.1 Research registration

This systematic review and meta-analysis was conducted in accordance with the Cochrane Handbook for Systematic Reviews of Interventions (version 6.3, 2022 update) and the Preferred Reporting Items for Systematic Reviews and Meta-Analyses (PRISMA) 2020 statement (Moher et al., 2009; Page et al., 2021) (Supplementary Material S1). The study protocol was registered in the International Prospective Register of Systematic Reviews (PROSPERO; registration number CRD420251009897).

### 2.2 Databases and search strategies

We systematically searched eight Chinese and English databases from inception to December 2024: PubMed, Cochrane Library, Embase, Web of Science, China National Knowledge Infrastructure (CNKI), Wanfang Data, China Science and Technology Journal Database (VIP), and China Biomedical Literature Database (CBM). ClinicalTrials.gov and the Chinese Clinical Trial Registry (ChiCTR) were also searched for ongoing studies. Search terms combined subject headings and free-text words, including “Yupingfeng Powder,” “Yupingfengsan,” “Yu-Ping-Feng-San,” “Rhinitis, Allergic,” and “Allergic Rhinitis.” Detailed strategies for each database are provided in Supplementary Material S1.

### 2.3 Inclusion criteria

Study type: Randomized controlled trials (RCTs) published in English or Chinese.

Participants: Patients diagnosed with allergic rhinitis, without restrictions on age, sex, or ethnicity.

Interventions: The experimental group received YPFS (original or modified formula), in any dosage form (e.g., decoction, granules), alone or combined with conventional pharmacotherapy. The control group received conventional pharmacotherapy or placebo. If both groups used conventional pharmacotherapy, the regimen had to be identical between groups.

Primary Outcomes: Total Nasal Symptom Score (TNSS), serum IgE level, overall effective rate, and adverse event rate. Secondary: Th1/Th2 ratio, Treg/Th17 ratio, IL-4, IL-6, and relapse rate.

### 2.4 Exclusion criteria

Study types: We excluded non-RCTs (e.g., case-control, cohort, cross-sectional studies, case reports, reviews, expert opinion).

Participants: Patients with acute sinusitis, acute conjunctivitis, active asthma, organic nasal lesions, or pregnant/lactating women.

Interventions: Multi-modal therapies in which YPFS was not the primary intervention (e.g., other TCM formulas, acupuncture, moxibustion, massage, acupoint injections). Control groups receiving treatments other than conventional pharmacotherapy or placebo.

Outcomes: studies without extractable data, or those unavailable in full text after author contact. For duplicate publications, the version with more complete data was retained.

### 2.5 Composition standards and botanical verification

YPFS is a multi-herbal formulation. This study adhered to botanical medicine reporting standards (Heinrich et al., 2022; GA-online, 2024) to ensure accurate and traceable documentation. The classical formula comprises Astragali mongholici radix, Atractylodis macrocephalae rhizoma, and Saposhnikoviae radix (Table 1). Modified YPFS includes these core herbs with additional botanicals (listed with scientific names in Supplementary Material S5). While most studies reported composition, some lacked details on processing methods or proportions. Commercial YPFS granules (Chinese Pharmacopoeia standard: Z10930036) were also included. For each trial, we documented formulation details and dosage forms, and verified the taxonomy of core herbs through Kew Royal Botanic Gardens resources (Royal Botanic Gardens, K, 2024; Royal Botanic Gardens, K, 2025).

**TABLE 1:** Basic information about YPFS.

Composition	Scientific name (authority)	Family	Pharmacopeial drug name	Source
Huang qi	Astragalus mongholicus Bunge	Fabaceae	astragali mongholici radix	European Pharmacop., 7th edn. (2012)
Bai zhu	Atractylodes macrocephala Koidz	Asteraceae	atractylodis macrocephalae rhizoma	European Pharmacop., 7th edn. (2012) Pharmacop. of China (2010) Pharmacop. of China (2015) Taiwan Herbal Pharmacop. 3rd Chinese ed. (MOHW, 2018)
Fang feng	Saposhnikovia divaricate (Turcz. ex Ledeb.) Schischk	Apiaceae	saposhnikoviae radix	Japanese Pharmacop., 15th edn. (2006) Japanese Pharmacop., 16th edn. (2012) Japanese Pharmacop., 17th edn. (2016–2019) Korean Pharmacop., 10th edn. (MFDS, 2012) Korean Pharmacop., 9th edn. (2007) Pharmacop. of China (2010) Pharmacop. of China (2015) Taiwan Herbal Pharmacop. 3rd Chinese ed. (MOHW, 2018)

### 2.6 Literature screening and data extraction

Search results were imported into EndNote 21. Two reviewers (Yumei Tang and Yangziting Bu) independently screened records in three stages: duplicate removal, title/abstract review, and full-text assessment based on eligibility criteria. Disagreements were resolved through discussion with a third reviewer (Jili Xu). Data extracted included author, year, study design, diagnostic criteria, sample size, sex, mean age, disease duration, treatment duration, interventions, and outcomes, using a pre-designed form with cross-checking.

### 2.7 Risk of bias assessment of included studies

Two reviewers independently evaluated study quality using the Cochrane Risk of Bias 2 (ROB-2) tool, covering: randomization process, deviations from intended interventions, missing outcome data, outcome measurement, and selective reporting. Each domain was rated as “low risk,” “some concerns,” or “high risk.” Discrepancies were resolved through discussion with a third reviewer.

### 2.8 Statistical analysis

Meta-analysis was performed using Review Manager 5.4 and Stata 18.0. For dichotomous variables, effect sizes were expressed as relative risk (RR) with 95% confidence intervals (CIs); for continuous variables, standardized mean difference (SMD) with 95% CIs was used to account for varying measurement units. Heterogeneity was assessed using the χ^2^ test and I^2^ statistic. A fixed-effects model was applied if I^2^ < 50% and p > 0.1; otherwise, a random-effects model was used, with subgroup analysis to explore sources of heterogeneity. Sensitivity analyses were conducted for all outcomes, including one based on risk of bias. For TNSS, IL-6, overall effectiveness, and adverse event rate (with ≥10 studies), publication bias was assessed using funnel plots and Egger’s test (p < 0.05 indicating bias). In such cases, the trim-and-fill method was applied to evaluate result robustness. Evidence quality was graded using GRADEpro.

### 2.9 Subgroup analysis

Predefined subgroup analyses were conducted to explore heterogeneity based on: Disease duration (>3 years, ≤3 years, unspecified), TCM pattern (Lung Qi Deficiency, Spleen–Lung Qi Deficiency, mixed/undifferentiated), Treatment duration (>4 weeks, ≤4 weeks), Age group (children, adults).

## 3 Results

### 3.1 Database search results and literature selection

**FIGURE 1:**
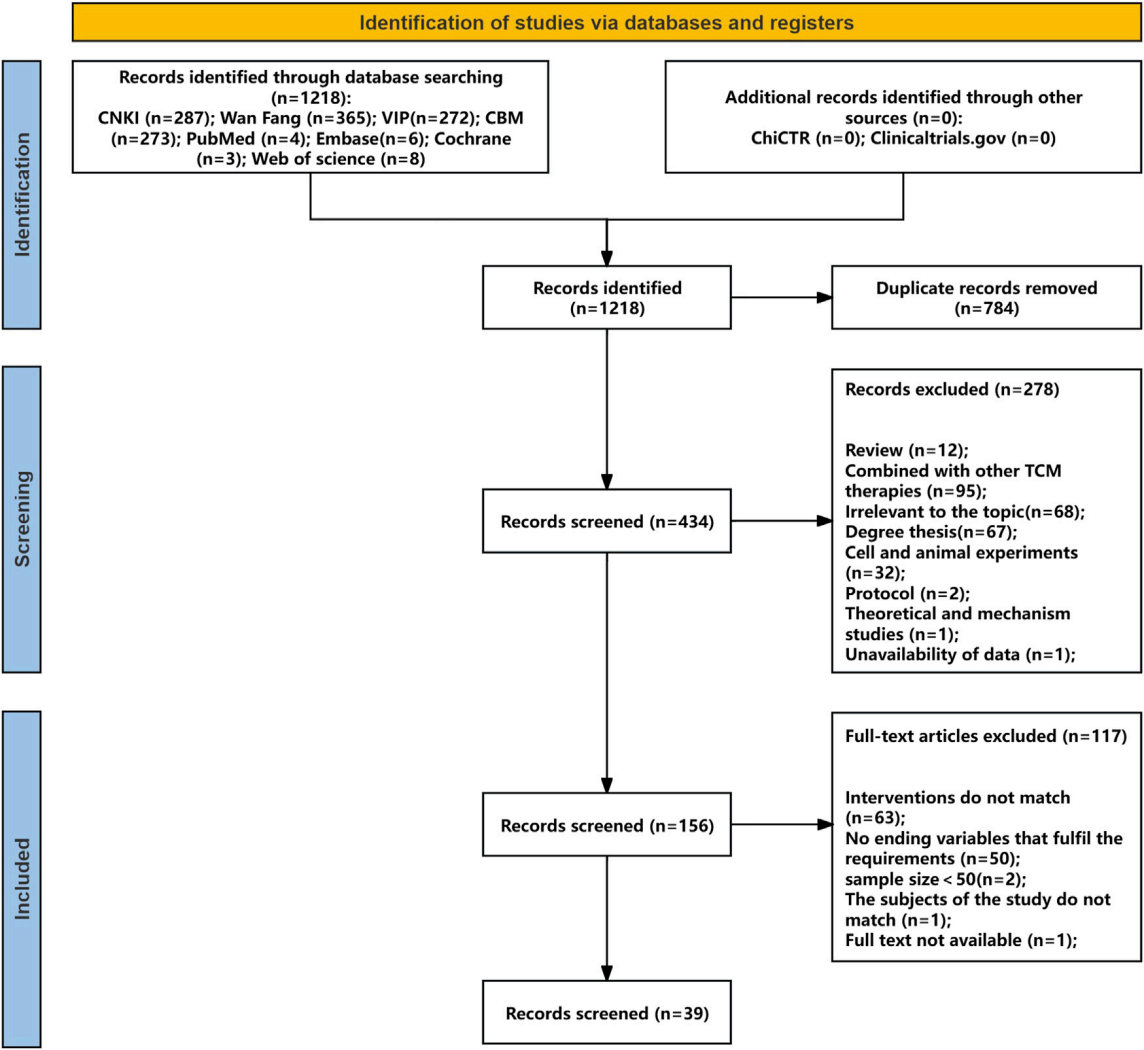
Flow diagram of studies selection process.

Searches of four Chinese databases, four English databases, and clinical trial registries identified 1,218 studies (1,197 from Chinese databases, 21 from English sources and registries). After removing 784 duplicates, 434 records remained. Screening of titles and abstracts excluded 278 studies. Full-text review of the remaining 156 studies, based on predefined inclusion and exclusion criteria, led to the exclusion of 117 articles for the following reasons: outcome variables not meeting requirements (n = 50), intervention not involving YPFS or combined with other TCM therapies (n = 63), sample size <50 (n = 2), mismatch in study population (n = 1), and unavailable full text (n = 1). In total, 39 randomized controlled trials (RCTs) met the eligibility criteria. The literature screening process is shown in Figure 1, with details provided in Supplementary Material S2,3.

### 3.2 Characteristics of the included studies

A total of 39 randomized controlled trials of YPFS for AR in China were included in this study, which were published between 2013 and 2024 (Chen, 2023; Chen, 2017; Cui and Chen, 2019; Deng et al., 2019; Dong, 2021; Du and Qian, 2017; Fang et al., 2017; Guan, 2013; Hong et al., 2023; Huang, 2024; Jiang, 2024; Jing et al., 2019; Kong et al., 2016; Li and Li, 2022; Li and Xuan, 2019; Li, 2023; Liao, 2019; Lin et al., 2024; Lv, 2024; Ma, 2017; Pan, 2024; Qiao, 2021; Qiu, 2017; Wan et al., 2021; Wang, 2023; Wang et al., 2021; Wang et al., 2019; Wang et al., 2022; Wu et al., 2016; Wu et al., 2018; Yang and Zhong, 2021; Yang and Yang, 2018; Yu et al., 2015; Fang et al., 2014; Zhang et al., 2023; Zhang, 2023; Zhang and Yuan, 2019; Zhao, 2020; Zheng, 2017) and involved a total of 4,578 patients with AR, including 2,308 in the trial group and 2,270 in the control group. Regarding diagnostic criteria, one study (Lv, 2024) used the World Health Organization (WHO) definition, 28 studies (Chen, 2023; Chen, 2017; Cui and Chen, 2019; Dong, 2021; Fang et al., 2017; Guan, 2013; Hong et al., 2023; Jiang, 2024; Kong et al., 2016; Li and Li, 2022; Liao, 2019; Lin et al., 2024; Pan, 2024; Qiu, 2017; Wan et al., 2021; Wang et al., 2021; Wang et al., 2019; Wang et al., 2022; Wu et al., 2016; Yang and Zhong, 2021; Yang and Yang, 2018; Yu et al., 2015; Fang et al., 2014; Zhang et al., 2023; Zhang, 2023; Zhang and Yuan, 2019; Zhao, 2020; Zheng, 2017) used the Chinese guideline diagnostic criteria, and no diagnostic criteria were reported in 10 studies (Deng et al., 2019; Du and Qian, 2017; Huang, 2024; Jing et al., 2019; Li and Xuan, 2019; Li, 2023; Ma, 2017; Qiao, 2021; Wang, 2023; Wu et al., 2018). The minimum duration of intervention was 14 days (Guan, 2013; Huang, 2024; Li, 2023; Wu et al., 2016), and the maximum was 3 months (Liao, 2019). Thirty-six studies (Chen, 2023; Chen, 2017; Cui and Chen, 2019; Deng et al., 2019; Dong, 2021; Du and Qian, 2017; Fang et al., 2017; Guan, 2013; Hong et al., 2023; Huang, 2024; Jiang, 2024; Jing et al., 2019; Kong et al., 2016; Li and Li, 2022; Li and Xuan, 2019; Li, 2023; Liao, 2019; Lin et al., 2024; Lv, 2024; Ma, 2017; Pan, 2024; Qiao, 2021; Qiu, 2017; Wan et al., 2021; Wang et al., 2021; Wang et al., 2019; Wang et al., 2022; Wu et al., 2016; Wu et al., 2018; Yang and Zhong, 2021; Yang and Yang, 2018; Yu et al., 2015; Fang et al., 2014; Zhang et al., 2023; Zhang and Yuan, 2019; Zheng, 2017) used the original YPFS formula, and three studies (Liao, 2019; Wang, 2023; Zhang, 2023; Zhao, 2020) used the YPFS modified formula, which were drug additions or subtractions based on the patient’s concomitant or concomitant symptoms. The composition of the original YPFS formula and its modified formulas is shown in Supplementary Material S5, and none of these studies reported quality control or chemical analyses of YPFS. Thirty-six studies (Chen, 2023; Chen, 2017; Cui and Chen, 2019; Deng et al., 2019; Dong, 2021; Du and Qian, 2017; Fang et al., 2017; Guan, 2013; Huang, 2024; Jiang, 2024; Jing et al., 2019; Kong et al., 2016; Li and Li, 2022; Li and Xuan, 2019; Liao, 2019; Lin et al., 2024; Lv, 2024; Ma, 2017; Pan, 2024; Qiao, 2021; Qiu, 2017; Wan et al., 2021; Wang, 2023; Wang et al., 2021; Wang et al., 2019; Wang et al., 2022; Wu et al., 2016; Wu et al., 2018; Yang and Zhong, 2021; Yang and Yang, 2018; Yu et al., 2015; Zhang et al., 2023; Zhang, 2023; Zhang and Yuan, 2019; Zhao, 2020; Zheng, 2017) used either the original YPFS formula or the modified formulas in combination with conventional pharmacotherapy in the test group and conventional pharmacotherapy in the control group; and three studies (Hong et al., 2023; Li, 2023; Fang et al., 2014) used the YPFS modified formulas alone in the test group and conventional pharmacotherapy in the control group. Thirty-nine randomized, controlled trials included in the studies had the following characteristics, are shown in Supplementary Material S4.

### 3.3 Risk of bias assessment

**FIGURE 2:**
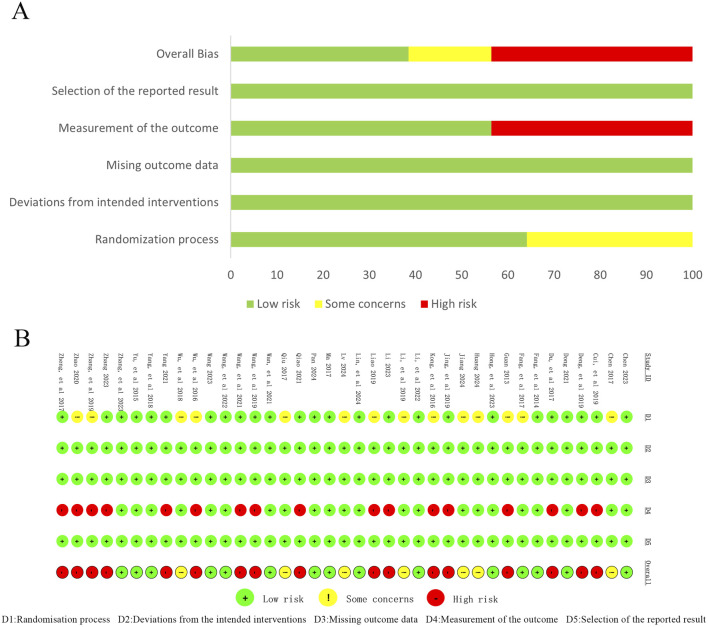
Risk of bias assessment for includedstudies. **(A)** Risk of bias graph; **(B)** Risk of bias summary.

Methodological quality was assessed using the Cochrane ROB-2 tool across five domains. Randomization process: 64% (25/39) low risk (random number tables used), 36% (14/39) some concerns (method not reported). Deviations from intended interventions, missing outcome data, selective reporting: all studies low risk. Outcome measurement: 56% (22/39) low risk, 44% (17/39) high risk due to unreported blinding of outcome assessors. Overall, the trials showed good control of bias in intervention adherence, data completeness, and reporting. However, insufficient reporting of randomization, allocation concealment, and blinding procedures remained a concern. Assessment results are presented in Figure 2.

### 3.4 Primary outcome indicators

#### 3.4.1 TNSS

##### 3.4.1.1 YPFS combined with conventional pharmacotherapy vs. conventional pharmacotherapy

**FIGURE 3:**
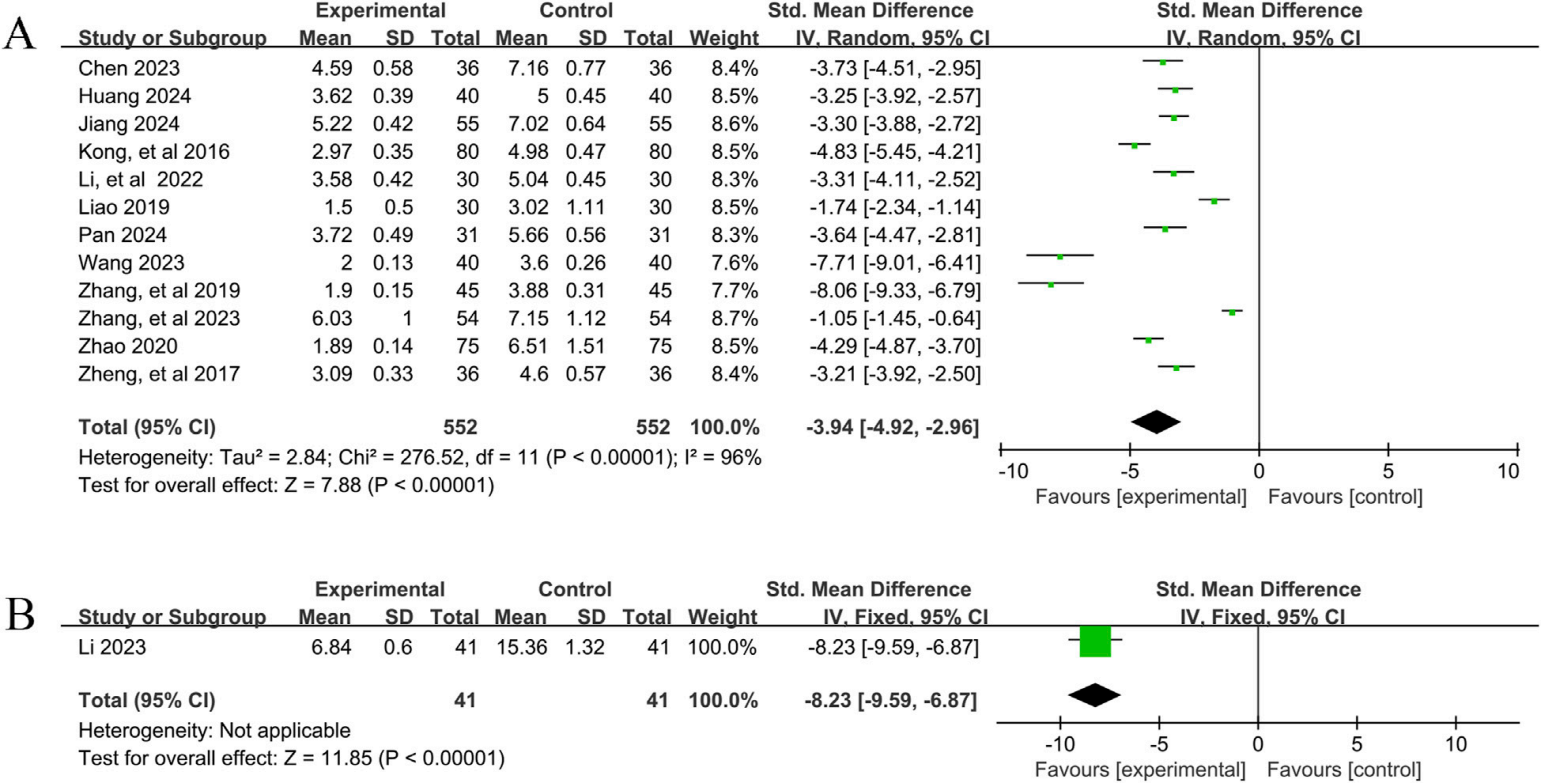
TNSS Forestplot for primary outcomes. **(A)** Combination therapy vs. Conventional pharmacotherapy; **(B)** YPFS treatment vs. Conventional pharmacotherapy.

A total of 12 studies (Chen, 2023; Huang, 2024; Jiang, 2024; Kong et al., 2016; Li and Li, 2022; Liao, 2019; Pan, 2024; Wang, 2023; Zhang, 2023; Zhang and Yuan, 2019; Zhao, 2020; Zheng, 2017) reported the efficacy of YPFS combined with conventional pharmacotherapy versus conventional pharmacotherapy for TNSS in 1,104 patients. A random-effects model was selected for statistical analysis based on the heterogeneity test (I^2^ = 96%, p < 0.00001). The results of the analysis showed that YPFS combined with conventional pharmacotherapy was effective in improving TNSS in AR patients compared with conventional pharmacotherapy, and the difference was statistically significant (SMD = −3.94, 95% CI [-4.92, −2.96], p < 0.00001, Figure 3A). However, due to the obvious heterogeneity between studies, we performed subgroup analyses based on different courses of disease, different TCM patterns, different durations, and different ages. In the results of subgroup analysis, there was no difference between different courses of disease (p = 0.21), different TCM patterns (p = 0.24), different duration (p = 0.99), and different ages (p = 0.54). The results of the subgroup analyses indicated that heterogeneity within the groups was not completely reduced, and therefore, these factors cannot be considered as the main source of heterogeneity at this time (Table 2, Supplementary Material S6.1). Sensitivity analyses showed similar amounts of combined effects, and the results were robust (Supplementary Material S7A).

**TABLE 2:** Subgroup analysis for TNSS, IgE and IL-6.

	Number of comparisons	Result: SMD/RR (95%CI)	p−value for overall effect	p−value for heterogeneity	I^2^ (%)	p−value for subgroup difference
TNSS						
All comparisons	12	−3.94 [-4.92, −2.96]	<0.00001	<0.00001	96	
Course of disease						0.21
>3 years	7	−4.56 [-5.93, -3.19]	<0.00001	<0.00001	96	
≤3 years	3	−3.01 [-5.27, −0.75]	0.009	<0.00001	98	
unspecified	2	−3.27 [-3.79, -2.76]	<0.00001	0.90	0	
TCM pattern						0.24
Lung Qi Deficiency Pattern	4	−3.62 [-5.80, -1.44]	0.001	<0.00001	98	
Lung-Spleen Qi Deficiency Pattern	3	−3.39 [-3.79, -2.98]	<0.00001	0.79	0	
Mixed/Undifferentiated	5	−4.53 [-5.80,-3.26]	<0.00001	<0.00001	93	
Duration						0.99
>4 weeks	5	−3.95 [-5.49, -2.42]	<0.00001	<0.00001	94	
≤4 weeks	7	−3.94 [-5.31, -2.57]	<0.00001	<0.00001	97	
Age						0.54
Child	1	−4.29 [-4.87,-3.70]	<0.00001	-	-	
Adult	11	−3.91 [-4.97, -2.85]	<0.00001	<0.00001	96	
IgE						
All comparisons	8	−2.11 [-2.53, -1.69]	<0.00001	<0.00001	84	
Course of disease						<0.0001
>3 years	4	−2.01 [-2.25, -1.78]	<0.00001	0.59	0	
≤3 years	1	−0.92 [-1.40, -0.43]	0.0002	-	-	
unspecified	3	−2.74 [-3.75, -1.72]	<0.00001	<0.0001	90	
TCM pattern						0.38
Lung Qi Deficiency Pattern	2	−1.86 [-2.24, -1.48]	<0.00001	0.64	0	
Lung-Spleen Qi Deficiency Pattern	1	−2.27 [-2.75, -1.79]	<0.00001	-	-	
Mixed/Undifferentiated	5	−2.20 [-2.87, -1.53]	<0.00001	<0.00001	90	
Duration						0.40
>4 weeks	2	−2.55 [-3.81, -1.30]	<0.0001	0.008	86	
≤4 weeks	6	−1.99 [-2.45, -1.52]	<0.00001	<0.00001	85	
Age						0.95
Child	2	−2.05 [-4.32, 0.22]	0.08	<0.00001	96	
Adult	6	−2.13 [−2.45, -1.81]	<0.00001	0.01	65	
IL-6						
All comparisons	10	−2.21 [-2.88, -1.53]	<0.00001	<0.00001	95	
Course of disease						0.48
>3 years	5	−2.29 [-3.06, -1.52]	<0.00001	<0.00001	92	
≤3 years	1	−1.72 [-2.27, -1.18]	<0.00001	-	-	
unspecified	4	−2.18 [-3.72, -0.64]	0.006	<0.00001	97	
TCM pattern						<0.00001
Lung Qi Deficiency Pattern	1	-4.79[-5.71, -3.88]	<0.00001	0.03	70	
Lung-Spleen Qi Deficiency Pattern	3	-1.37[-1.91, -0.83]	<0.00001	-	-	
Mixed/Undifferentiated	6	-2.25[-3.10, -1.39]	<0.00001	<0.00001	95	
Duration						0.006
>4 weeks	4	−1.37 [-1.71, -1.04]	<0.00001	0.22	31	
≤4 weeks	6	−2.76 [-3.69, -1.82]	<0.00001	<0.00001	96	
Age						0.02
Child	2	−1.37 [-1.79, -0.96]	<0.00001	0.30	8	
Adult	8	−2.41 [-3.20, -1.62]	<0.00001	<0.00001	95	

##### 3.4.1.2 Comparison of YPFS with conventional pharmacotherapy

One study (Li, 2023), including 82 patients with AR, reported that YPFS treatment was more effective than conventional pharmacotherapy in reducing TNSS after 14 days, and the difference was statistically significant. (SMD = −8.23, 95% CI −9.59, −6.87], p < 0.00001, Figure 3B).

#### 3.4.2 IgE

##### 3.4.2.1 YPFS combined with conventional pharmacotherapy vs. conventional pharmacotherapy

**FIGURE 4:**
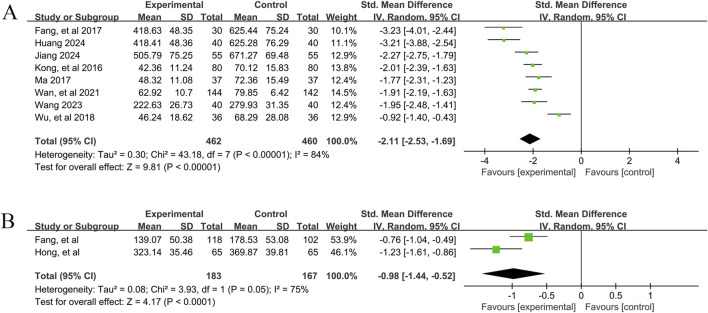
IgE Forestplot forprimary outcomes. **(A)** Combination therapy vs. Conventional pharmacotherapy; **(B)** YPFS treatment vs. Conventional pharmacotherapy.

In total, eight studies (Fang et al., 2017; Huang, 2024; Jiang, 2024; Kong et al., 2016; Ma, 2017; Wan et al., 2021; Wang, 2023; Wu et al., 2018) reported the efficacy of YPFS combined with conventional pharmacotherapy versus conventional pharmacotherapy on IgE levels in 922 patients. A random-effects model was selected for statistical analysis based on the heterogeneity test (I^2^ = 84%, p < 0.00001). The results of the analysis showed that YPFS combined with conventional pharmacotherapy was effective in improving IgE levels in AR patients compared with conventional pharmacotherapy, and the difference was statistically significant (SMD = −2.11, 95% CI [-2.53, −1.69], p < 0.00001, Figure 4A). However, due to the significant heterogeneity among studies, we performed subgroup analyses based on different courses of disease, different TCM patterns, different durations, and different ages. In the results of subgroup analysis, there were no differences between different TCM patterns (p = 0.38), different durations (p = 0.40), and different ages (p = 0.95). Differences existed by courses of disease (p < 0.0001). However, heterogeneity within the groups was not completely reduced, so these factors cannot be considered as the main source of heterogeneity at this time (Table 2, Supplementary Material S6.2). In addition, sensitivity analyses were performed to assess whether the combined effect sizes might have been significantly influenced by a single study. Sensitivity analyses indicated that the combined effect sizes were similar and that the results were robust (Supplementary Material S7B).

##### 3.4.2.2 Comparison of YPFS with conventional pharmacotherapy

A total of 2 studies (Fang et al., 2014; Hong et al., 2023) reported the efficacy of YPFS combined with conventional pharmacotherapy versus conventional pharmacotherapy on IgE levels in 350 patients. A random-effects model was selected for statistical analysis based on the test for heterogeneity (I^2^ = 75%, p = 0.05). The results of the analysis showed that YPFS was effective in improving IgE levels in AR patients compared with conventional pharmacotherapy, and the difference was statistically significant (SMD = −0.98, 95% CI [-1.44, −0.52], p < 0.0001, Figure 4B). Sensitivity analyses indicated that the combined effect sizes were similar and that the results were robust (Supplementary Material S7C).

#### 3.4.3 Overall effective rate

##### 3.4.3.1 YPFS combined with conventional pharmacotherapy vs. conventional pharmacotherapy

**FIGURE 5:**
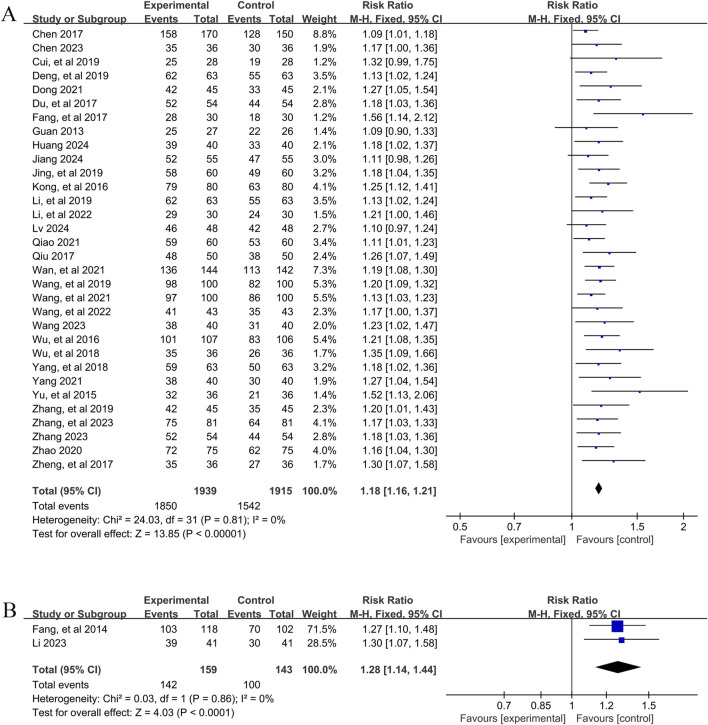
Overall effectiveness rate Forestplot for primary outcomes. **(A)** Combination therapy vs. Conventional pharmacotherapy; **(B)** YPFS treatment vs. Conventional pharmacotherapy.

A total of 32 studies (Chen, 2023; Chen, 2017; Cui and Chen, 2019; Deng et al., 2019; Dong, 2021; Du and Qian, 2017; Fang et al., 2017; Guan, 2013; Huang, 2024; Jiang, 2024; Jing et al., 2019; Kong et al., 2016; Li and Li, 2022; Li and Xuan, 2019; Lv, 2024; Qiao, 2021; Qiu, 2017; Wan et al., 2021; Wang, 2023; Wang et al., 2021; Wang et al., 2019; Wang et al., 2022; Wu et al., 2016; Wu et al., 2018; Yang and Zhong, 2021; Yang and Yang, 2018; Yu et al., 2015; Zhang et al., 2023; Zhang, 2023; Zhang and Yuan, 2019; Zhao, 2020; Zheng, 2017) involving 3,854 patients reported the overall efficacy of YPFS combined with conventional pharmacotherapy versus conventional pharmacotherapy. According to the heterogeneity test (I^2^ = 0%, p = 0.81), a fixed-effects model was selected for statistical analysis. The results of the analysis showed that the total effective rate of YPFS combined with conventional pharmacotherapy was greater than that of conventional pharmacotherapy, and the difference was statistically significant (RR = 1.18, 95% CI [1.16, 1.21], p < 0.00001, Figure 5A). Sensitivity analyses indicated that the results were robust (Supplementary Material S7D).

##### 3.4.3.2 Comparison of YPFS with conventional pharmacotherapy

A total of 2 studies (Fang et al., 2014; Li, 2023) involving 302 patients reported the overall efficacy of YPFS combined with conventional pharmacotherapy vs. conventional pharmacotherapy. A fixed-effects model was selected for statistical analysis based on the heterogeneity test (I^2^ = 0%, p = 0.86). The results of the analysis showed that the total effective rate of YPFS was greater than that of conventional pharmacotherapy, and the difference was statistically significant (RR = 1.28, 95% CI [1.14, 1.44], p < 0.0001, Figure 5B). Sensitivity analysis indicated that the results were robust (Supplementary Material S7E).

#### 3.4.4 Adverse event rate

##### 3.4.4.1 YPFS combined with conventional pharmacotherapy vs. conventional pharmacotherapy

**FIGURE 6:**
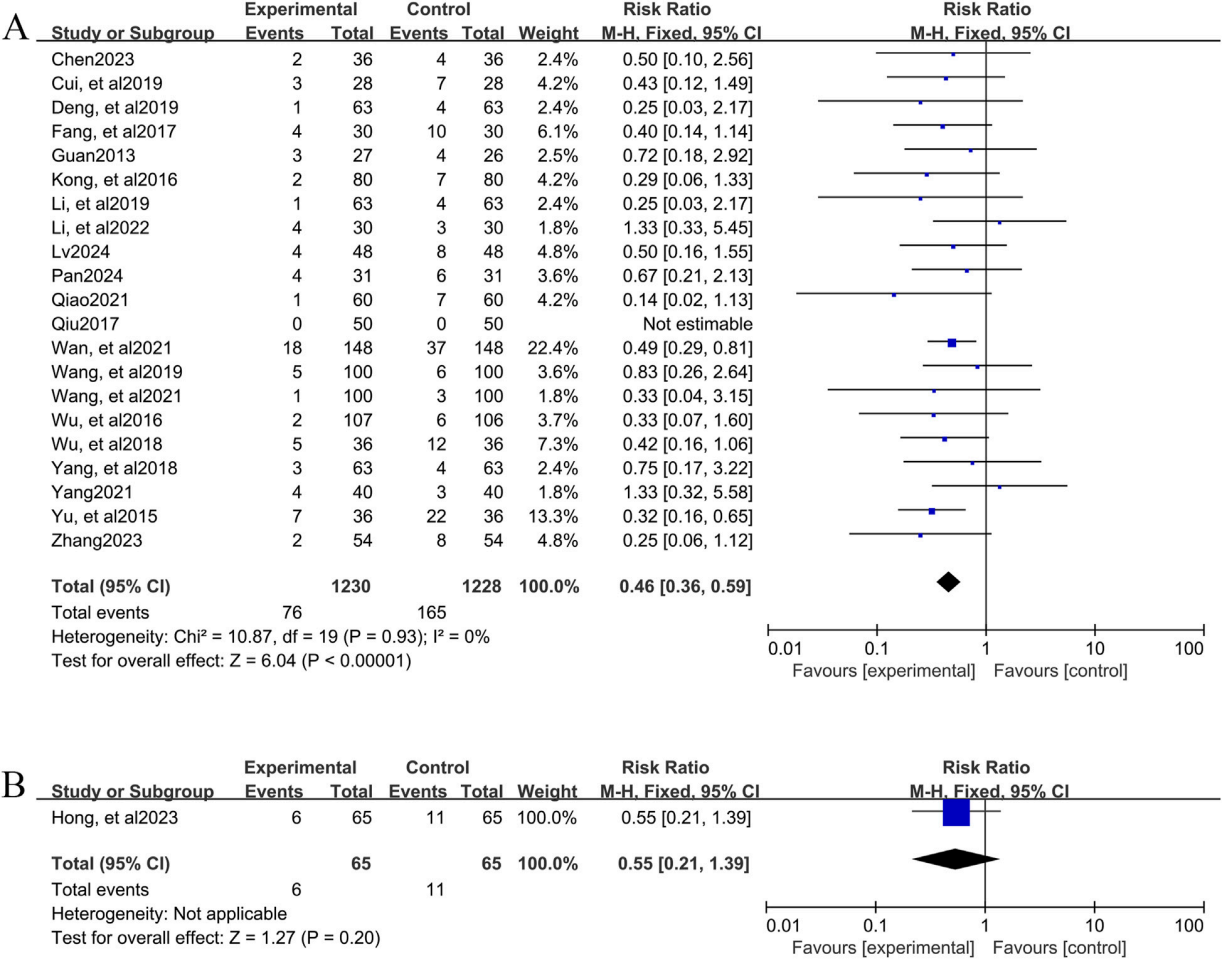
Adverse event rate Forestplot for primary outcomes. **(A)** Combination therapy vs. Conventional pharmacotherapy; **(B)** YPFS treatment vs. Conventional pharmacotherapy.

A total of 20 studies (Chen, 2023; Cui and Chen, 2019; Deng et al., 2019; Fang et al., 2017; Guan, 2013; Kong et al., 2016; Li and Li, 2022; Li and Xuan, 2019; Lv, 2024; Pan, 2024; Qiao, 2021; Wan et al., 2021; Wang et al., 2021; Wang et al., 2019; Wu et al., 2016; Wu et al., 2018; Yang and Zhong, 2021; Yang and Yang, 2018; Yu et al., 2015; Zhang, 2023), involving 2,458 patients, reported the incidence of adverse events for YPFS combined with conventional pharmacotherapy versus conventional pharmacotherapy. According to the heterogeneity test (I^2^ = 0%, p = 0.93), a fixed-effects model was selected for statistical analysis. The results of the analysis showed that the incidence of adverse events was smaller in YPFS combined with conventional pharmacotherapy compared to, and the difference was statistically significant (RR = 0.46, 95% CI [0.36, 0.59], p < 0.00001, Figure 6A). Sensitivity analyses indicated that the results were robust (Supplementary Material S7F).

##### 3.4.4.2 Comparison of YPFS with conventional pharmacotherapy

One study (Hong et al., 2023), including 130 AR patients, reported 6 (9.23%) adverse events in the YPFS combined with conventional pharmacotherapy group and 11 (16.92%) adverse events in the control group; however, no specific adverse reactions were stated. The results of the analysis showed that the incidence of adverse events in YPFS was less than that of conventional pharmacotherapy, and the difference was statistically significant. (RR = 0.55, 95% CI [0.21, 1.39], p = 0.20, Figure 6B).

Across the 39 included studies, reporting of adverse events varied considerably. Fifteen studies did not address adverse event monitoring or outcomes, while 24 provided relevant data. Of these, three explicitly stated that no adverse events occurred during treatment, and 21 reported at least one adverse event (Supplementary Material S11). Events linked to YPFS were generally mild to moderate, most often involving the digestive system (e.g., constipation, abdominal discomfort) or the nervous system (e.g., dizziness). Serious adverse events were rare. In head-to-head comparisons, YPFS monotherapy was consistently associated with fewer adverse events than conventional Western medications alone. When combined with Western treatments, some studies indicated that YPFS might help reduce certain medication-related side effects.

### 3.5 Secondary outcome indicators

#### 3.5.1 Th1/Th2

##### 3.5.1.1 YPFS combined with conventional pharmacotherapy vs. conventional pharmacotherapy

**FIGURE 7:**
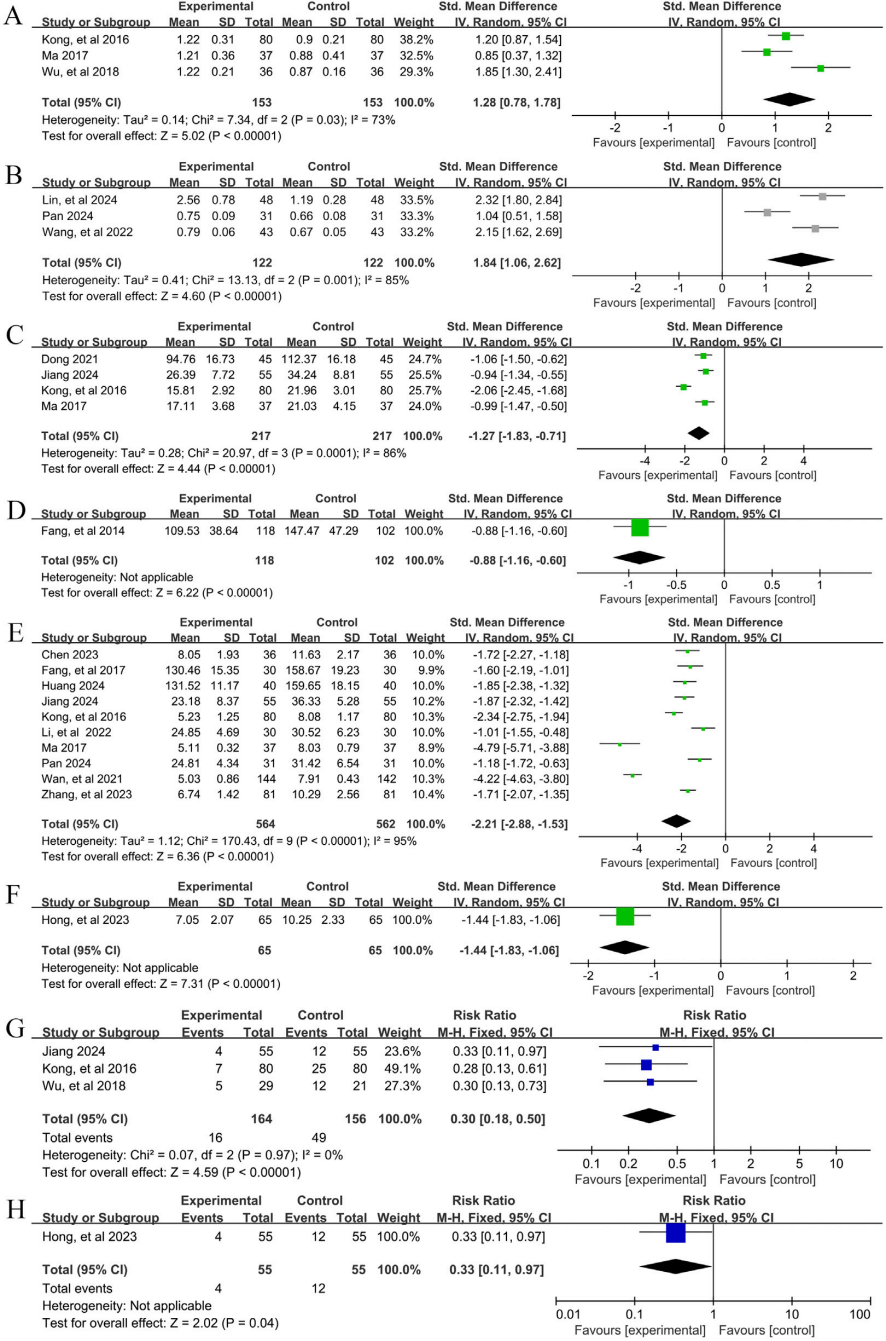
Secondary outcome indicators Forestplot for primary outcomes. **(A)** Th1/Th2 (Combination therapy vs. Conventional pharmacotherapy); **(B)** Treg/Th17 (Combination therapy vs. Conventional pharmacotherapy); **(C)** IL-4 YPFS treatment vs. Conventional pharmacotherapy; **(D)** IL-4 (Combination therapy vs. Conventional pharmacotherapy); **(E)** IL-6 (Combination therapy vs. Conventional pharmacotherapy); **(F)** IL-6 (Combination therapy vs. Conventional pharmacotherapy); **(G)** Relapse rate (Combination therapy vs. Conventional pharmacotherapy); **(H)** Relapse rate (YPFS treatment vs. Conventional pharmacotherapy).

In total, three studies (Kong et al., 2016; Ma, 2017; Wu et al., 2018) reported the efficacy of YPFS combined with conventional pharmacotherapy versus conventional pharmacotherapy on Th1/Th2, involving 306 patients. A random-effects model was selected for statistical analysis based on the heterogeneity test (I^2^ = 73%, p = 0.03). The results of the analysis showed that YPFS combined with conventional pharmacotherapy was effective in improving Th1/Th2 in AR patients compared with conventional pharmacotherapy, and the difference was statistically significant (SMD = 1.28, 95% CI [0.78, 1.78], p < 0.00001, Figure 7A). Subgroup analyses were not performed because the number of studies was too small to determine the source of heterogeneity by subgroup analysis. Sensitivity analyses indicated that the results were robust (Supplementary Material S7G).

#### 3.5.2 Treg/Th17

##### 3.5.2.1 YPFS combined with conventional pharmacotherapy vs. conventional pharmacotherapy

In total, three studies (Lin et al., 2024; Pan, 2024; Wang et al., 2022) reported the efficacy of YPFS combined with conventional pharmacotherapy versus conventional pharmacotherapy for Treg/Th17, involving a total of 244 patients. A random-effects model was selected for statistical analysis based on the heterogeneity test (I^2^ = 85%, p = 0.001). The results of the analysis showed that YPFS combined with conventional pharmacotherapy was effective in improving Treg/Th17 in AR patients compared with conventional pharmacotherapy, and the difference was statistically significant (SMD = 1.84, 95% CI [1.06, 2.62], p < 0.00001, Figure 7B). Subgroup analyses were not performed because the number of studies was too small to determine the source of heterogeneity by subgroup analysis. Sensitivity analyses indicated that the results were robust (Supplementary Material S7H).

#### 3.5.3 IL-4

##### 3.5.3.1 YPFS combined with conventional pharmacotherapy vs. conventional pharmacotherapy

In total, four studies (Dong, 2021; Jiang, 2024; Kong et al., 2016; Ma, 2017) reported the efficacy of YPFS combined with conventional pharmacotherapy versus conventional pharmacotherapy on IL-4 levels in 434 patients. A random-effects model was selected for statistical analysis based on the heterogeneity test (I^2^ = 86%, p = 0.0001). The results of the analysis showed that YPFS combined with conventional pharmacotherapy was effective in improving IL-4 levels in AR patients compared with conventional pharmacotherapy, and the difference was statistically significant (SMD = −1.27, 95% CI [-1.83, −0.71], p < 0.00001, Figure 7C). Subgroup analyses were not performed because the number of studies was too small to determine the source of heterogeneity by subgroup analysis. Sensitivity analyses indicated that the results were robust (Supplementary Material S7I).

##### 3.5.3.2 Comparison of YPFS with conventional pharmacotherapy

One study (Fang et al., 2014), including 220 AR patients, reported that YPFS treatment was more effective in reducing IL-4 than conventional pharmacotherapy after 1 month, and the difference was statistically significant. (SMD = −0.88, 95% CI [-1.16, −0.60], p < 0.00001, Figure 7D).

#### 3.5.4 IL-6

##### 3.5.4.1 YPFS combined with conventional pharmacotherapy vs. conventional pharmacotherapy

In total, 10 studies (Chen, 2023; Fang et al., 2017; Huang, 2024; Jiang, 2024; Kong et al., 2016; Li and Li, 2022; Ma, 2017; Pan, 2024; Wan et al., 2021; Zhang et al., 2023) reported the efficacy of YPFS combined with conventional pharmacotherapy versus conventional pharmacotherapy on IL-6 levels, involving a total of 1,126 patients. A random-effects model was selected for statistical analysis based on the heterogeneity test (I^2^ = 95%, p < 0.00001). The results of the analysis showed that YPFS combined with conventional pharmacotherapy was effective in improving IL-6 levels in AR patients compared with conventional pharmacotherapy, and the difference was statistically significant (SMD = −2.21, 95% CI [-2.88, −1.53], p < 0.0001, Figure 7E). However, due to the obvious heterogeneity among different studies, we performed subgroup analyses based on different courses of disease, different TCM patterns, different durations, and different ages. In the results of subgroup analysis, there were no differences between different courses of disease (p = 0.48), different TCM patterns (p < 0.00001), and different durations (p = 0.006). Differences were found between different ages (p = 0.02); however, heterogeneity within groups was not completely reduced, so these factors cannot be considered as a major source of heterogeneity at this time (Table 2, Supplementary Material S6.3). Sensitivity analyses indicated that the results were robust (Supplementary Material S7J).

##### 3.5.4.2 Comparison of YPFS with conventional pharmacotherapy

One study (Hong et al., 2023), including 130 AR patients, reported that YPFS was more effective than Conventional pharmacotherapy in lowering IL-6 after 8 weeks of treatment, and the difference was statistically significant. (SMD = −1.44, 95% CI [-1.83, −1.06], p < 0.00001, Figure 7F).

#### 3.5.5 Relapse rate

One study (Hong et al., 2023) explicitly defines the recurrence rate as: if RCAT ≤21 points, the condition is uncontrolled and in a relapse state; recurrence rate = (number of AR patients experiencing relapse within 6 months/total number of patients) × 100%. However, the other three studies (Jiang, 2024; Kong et al., 2016; Wu et al., 2018) did not provide a clear definition for the recurrence rate.

##### 3.5.5.1 YPFS combined with conventional pharmacotherapy vs. conventional pharmacotherapy

A total of 3 studies (Jiang, 2024; Kong et al., 2016; Wu et al., 2018), involving 320 patients, reported the recurrence rate of YPFS combined with conventional pharmacotherapy versus conventional pharmacotherapy. A fixed-effects model was selected for statistical analysis based on the heterogeneity test (I^2^ = 0%, p = 0.97). The results of the analysis showed that the relapse rate of YPFS combined with conventional pharmacotherapy was smaller than that of conventional pharmacotherapy, and the difference was statistically significant (RR = 0.30, 95% CI [0.18, 0.50], p < 0.00001, Figure 7G). Sensitivity analysis showed that the results were robust (Supplementary Material S7K).

##### 3.5.5.2 Comparison of YPFS with conventional pharmacotherapy

One study (Hong et al., 2023) that included 130 AR patients reported a statistically significant difference in recurrence rates for YPFS compared with conventional pharmacotherapy, which was less than conventional pharmacotherapy. (RR = 0.33, 95% CI [0.11, 0.97], p = 0.04, Figure 7H).

### 3.6 Sensitivity analysis based on risk of bias

To evaluate the influence of methodological quality on pooled estimates, we conducted a prespecified sensitivity analysis excluding 17 studies rated “high risk” in ≥2 key bias domains (random sequence generation, allocation concealment, blinding). The remaining 22 studies were reanalyzed. Effect directions and statistical significance for TNSS, IgE, Treg/Th17, IL-4, IL-6, total effective rate, and adverse event incidence remained unchanged (Supplementary Material S8). Due to insufficient eligible studies (n < 2), no pooled estimate for Th1/Th2 could be obtained. These findings suggest that the main results are directionally stable despite methodological limitations.

### 3.7 Publication bias

**FIGURE 8:**
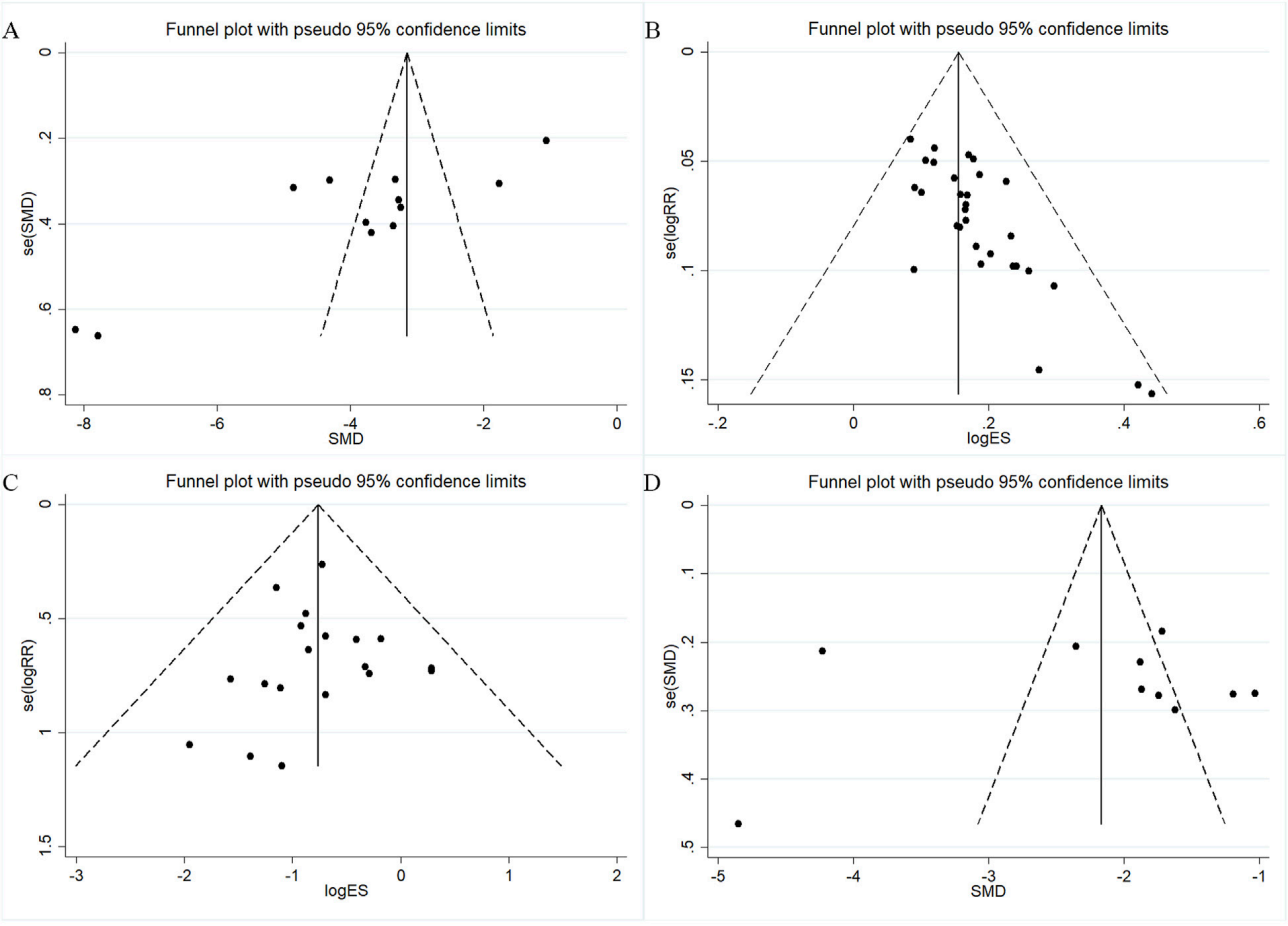
Funnel plots for assessing publication bias. **(A)** TNSS; **(B)** Overall effectiveness rate; **(C)** Adverse event rate; **(D)** IL-6.

Publication bias was assessed for outcomes with ≥10 studies using funnel plots and Egger’s test. For TNSS, the funnel plot was asymmetric (Figure 8A) and Egger’s test indicated significant bias (p = 0.001; Supplementary Material S9A). Trim-and-fill analysis imputed three studies after five iterations (Supplementary Material S10A), with minimal changes to effect size estimates. The overall effective rate also showed asymmetry (Figure 8B) and significant bias (p < 0.001; Supplementary Material S9B). After trim-and-fill correction, 10 studies were added (Supplementary Material S10B), again with only minor effect size changes. These results suggest that while some publication bias may exist, its influence on the pooled outcomes is small. For adverse event incidence, the funnel plot showed slight asymmetry (Figure 8C), but Egger’s test was not significant (p = 0.811; Supplementary Material S9C). Trim-and-fill adjustment added no substantial bias, and effect estimates changed minimally (SMD: 3.978 before vs. −4.658 after correction). IL-6 studies displayed symmetric funnel plots (Figure 8D) and no significant bias (p = 0.869; Supplementary Material S9D). Overall, the analyses indicate that potential publication bias has little impact on the robustness of results.

### 3.8 Assessment of the quality of evidence

Using the GRADEpro framework, all outcomes were rated as low to very low certainty (Supplementary Material S12). The main reasons for downgrading included unclear randomization procedures, absence of allocation concealment, and lack of blinding in all trials. These limitations highlight the need for large-scale, rigorously designed RCTs with standardized methodologies to confirm the clinical efficacy and safety of YPFS for allergic rhinitis.

## 4 Discussion

### 4.1 Main results of this research

Systematic reviews and meta-analyses are essential tools in evidence-based medicine, offering high-level evidence to guide clinical decision-making. In this study, we evaluated the efficacy and safety of YPFS for AR through a comprehensive systematic review and meta-analysis. A total of 39 studies, identified from both Chinese and English databases, were included following strict eligibility criteria. Our analysis assessed multiple outcomes, including modulation of immune homeostasis, inflammatory marker regulation, overall clinical effectiveness, adverse event incidence, and recurrence rates. Subgroup analyses were conducted to explore potential sources of heterogeneity, such as disease duration, TCM pattern differentiation, treatment length, and patient age. Sensitivity analyses tested the robustness of findings, while publication bias was examined using funnel plots and Egger’s test. The certainty of evidence was graded using the GRADEpro framework. The results suggest that YPFS combined with conventional pharmacotherapy outperforms conventional Western therapy alone in improving Total Nasal Symptom Score (TNSS), modulating Th1/Th2 and Treg/Th17 balance, and lowering serum IgE, IL-4, and IL-6 levels. As monotherapy, YPFS also improved TNSS and reduced IgE, IL-4, and IL-6 levels compared with Western medicine alone. Both monotherapy and combination therapy improved overall clinical effectiveness and reduced relapse rates. However, evidence on the effects of YPFS monotherapy on Th1/Th2, Treg/Th17, and IL-4 is limited due to the small number of relevant trials. Adverse events linked to YPFS were generally mild and infrequent, but reporting was inconsistent—many studies did not monitor or report safety data. Consequently, current evidence is insufficient to draw firm conclusions about the long-term safety profile of YPFS. Although publication bias was detected for some outcomes, trim-and-fill adjustments showed minimal impact on overall effect estimates.

Despite strict screening, substantial heterogeneity remained in most analyses. Likely contributors include methodological differences across studies, variations in patient constitution and symptom patterns, seasonal and regional influences on disease presentation, and modifications to TCM prescriptions in clinical practice. Patient age ranges were broad, with children, adults, and older adults potentially responding differently due to variations in immune status and drug tolerance. Subgroup and sensitivity analyses, along with GRADE assessments, were used to explore these factors and verify the robustness of the main results.

Importantly, all included RCTs shared critical methodological weaknesses: allocation concealment was never reported, and blinding procedures were either absent or poorly described. These deficiencies led to “high risk” ratings in key bias domains and downgrading of the certainty of evidence for primary outcomes to low or very low. As such, the current efficacy findings should be interpreted with caution. High-quality, large-scale RCTs that follow international reporting standards (e.g., CONSORT) are urgently needed to confirm the therapeutic value and safety of YPFS for AR and to clarify its mechanisms of action.

### 4.2 Possible mechanisms of YPFS for AR treatment

The pathomechanism of allergic rhinitis (AR) involves a complex interplay of immune and inflammatory processes. Upon exposure to allergens such as pollen or dust mites, antigen-presenting cells in the nasal cavity capture and process the antigens, subsequently transmitting antigenic information to Th2 lymphocytes. Activated Th2 cells secrete cytokines including IL-4, IL-5, and IL-13, among which IL-4 and IL-13 induce B cells to differentiate into plasma cells and produce allergen-specific IgE, while IL-5 promotes eosinophil proliferation and activation. The generated IgE binds via its Fc segment to FcεRI receptors on mast cells and basophils, establishing a sensitized state. Upon re-exposure to the allergen, cross-linking of two adjacent IgE molecules triggers mast cell and basophil degranulation, releasing preformed mediators such as histamine and proteases, followed by the synthesis and release of additional inflammatory mediators. These bioactive substances act on nerve, epithelial, and glandular cells, producing clinical symptoms including sneezing, rhinorrhea, nasal pruritus, and congestion (Bernstein et al., 2024; Bousquet et al., 2020).

The intricate pathogenesis of AR and the incomplete understanding of the protective mechanisms of YPFS present major challenges to its clinical application. Based on current evidence, the possible mechanisms of YPFS in AR treatment can be summarized as follows.

#### 4.2.1 Regulation of immune homeostasis

The immunomodulatory effects of YPFS on AR are characterized by multi-target, multi-level regulation of immune imbalance and the remodeling of immune homeostasis. Experimental studies demonstrate that YPFS can bidirectionally regulate Th1/Th2 and Th17/Treg cell subsets to correct immune deviation. It can upregulate Th1-specific transcription factor T-bet while inhibiting Th2-specific GATA-3, thereby promoting Th0 cell differentiation toward Th1 and suppressing Th2 polarization. This leads to increased secretion of IFN-γ, reduced levels of Th2-type cytokines such as IL-4, IL-5, and IL-13, and attenuation of IgE-mediated mast cell degranulation and eosinophil infiltration (Wang and Hu, 2016; Chen et al., 2021; Bin et al., 2024; Huang et al., 2022). Concurrently, YPFS suppresses the pro-inflammatory activity of Th17 cells by decreasing serum IL-17A levels and enhancing IL-10 and TGF-β1 expression, thereby promoting CD4^+^CD25+Foxp3+ Treg cell differentiation, restoring the Treg/Th17 balance, and strengthening immunosuppressive function (Zhou et al., 2020; Jia et al., 2016). Furthermore, YPFS enhances immunoregulation by restoring IL-10 expression in regulatory B cells (Bregs) through inhibition of Bcl2L12, acting synergistically across multiple pathways to ameliorate immune dysfunction in AR and exert therapeutic benefits (Zhou et al., 2019).

#### 4.2.2 Intervention with inflammatory mediators

YPFS exhibits significant anti-inflammatory activity in the treatment of AR. At the transcription factor and signaling pathway levels, it inhibits Th2 cell differentiation and associated inflammatory cascades by downregulating the expression of Th2-specific transcription factor GATA-3 in nasal mucosal tissues (Long et al., 2014), and simultaneously suppresses NF-κB p65 protein and gene expression to modulate the TLR4/NF-κB signaling pathway. This blockade, which is dose-dependent, prevents transcriptional activation of multiple inflammatory mediators, thereby alleviating tissue inflammatory injury (Lin and Huang, 2018). Regarding inflammatory mediator regulation, YPFS markedly reduces pro-inflammatory factors such as IL-6 and TNF-α in peripheral plasma, peritoneal mast cells, and nasal mucosa (Zhong-lin et al., 2014a), while decreasing Th2 cytokines including IL-4, IL-5, and IL-13, inhibiting IgE synthesis, and attenuating IgE-mediated allergic responses. In parallel, it enhances anti-inflammatory cytokines such as IL-10 and IFN-γ, restoring the dynamic balance between pro- and anti-inflammatory mediators (Lin and Huang, 2018). Moreover, YPFS can target epidermal growth factor receptor (EGFR), mitogen-activated protein kinase 1 (MAPK1), and protein kinase B (AKT1), thereby inhibiting downstream inflammatory signaling, reducing mediator release, and mitigating pathological features including tissue remodeling, stromal edema, and eosinophil infiltration in the nasal mucosa, ultimately exerting anti-inflammatory therapeutic effects (Liu et al., 2022).

#### 4.2.3 Stabilization of mast cells

YPFS demonstrates a bidirectional regulatory effect on mast cells in AR, stabilizing their activity by upregulating IL-3 (a mast cell growth factor) and inhibiting G-CSF (a mast cell proliferation-promoting factor). This reduces mast cell degranulation and the release of inflammatory mediators such as histamine, trypsin-like proteases, and IL-13, thereby alleviating AR symptoms (Zhong-lin et al., 2014b). In murine AR models, YPFS treatment significantly improved nasal symptoms, reduced mast cell infiltration in the nasal mucosa, and lowered trypsin-like enzyme activity, plasma histamine, and IL-13 levels, indicating inhibition of mast cell recruitment, maturation, and the inflammatory responses they mediate (Lu et al., 2021; Zhong-lin et al., 2014c). Furthermore, YPFS reduces the proportion of CD117+FcεRI + double-positive bone marrow cells, suggesting suppression of mast cell differentiation and activation (Lu et al., 2021). Collectively, these findings indicate that YPFS ameliorates nasal mucosal inflammation and improves AR clinical symptoms by modulating mast cell differentiation, maturation, tissue infiltration, and degranulation.

#### 4.2.4 Inhibition of cellular pyroptosis

Cellular pyroptosis, a form of programmed cell death, promotes the release of large amounts of pro-inflammatory cytokines, contributing to inflammatory diseases. Inflammasomes, activated by various stimuli, can trigger pyroptosis; persistent activation of the NLRP3 inflammasome upregulates Caspase-1 and gasdermin D (GSDMD), leading to cytokine secretion, epithelial cell death, nasal mucosal damage, and epithelial barrier disruption (Yang et al., 2020). Experimental evidence shows that YPFS inhibits reactive oxygen species (ROS) accumulation and suppresses activation of the ROS/NLRP3/Caspase-1 axis in nasal mucosa, downregulating pyroptosis-associated proteins including NLRP3, cleaved Caspase-1, and cleaved GSDMD. This blocks pyroptosis-mediated inflammatory cascades, alleviates goblet cell hyperplasia, and mitigates mucosal histopathological damage (Wu et al., 2023).

### 4.3 Strengths and limitations of this study

In this study, we systematically evaluated the efficacy and safety of YPFS for AR and summarized its possible mechanisms of action, providing the most up-to-date and comprehensive evidence to date. Notably, this is the first meta-analysis to not only confirm the benefits of YPFS in improving TNSS but also to quantitatively assess its immunomodulatory effects on Th1/Th2 and Treg/Th17 balance and its influence on inflammatory mediators such as IL-4 and IL-6. These findings provide higher-level evidence for the immune-regulatory mechanisms proposed in traditional Chinese medicine. We distinguished between combination therapy and monotherapy through dual-path analyses, enabling clearer comparisons of YPFS as an adjunctive versus stand-alone treatment and generating more nuanced clinical evidence. Subgroup analyses and trim-and-fill adjustments were applied to address heterogeneity, with consistent results suggesting applicability in complex real-world treatment settings. This work also advances beyond the scope of traditional meta-analyses by including recurrence rates and safety outcomes, helping to fill the evidence gap on the long-term efficacy of traditional Chinese medicine interventions. Methodologically, the review adhered strictly to PRISMA standards, incorporated GRADE evidence grading, and applied sensitivity analyses to form a closed-loop quality appraisal, ensuring cautious interpretation and reducing the risk of misleading conclusions. Nevertheless, several methodological and evidence-level limitations must be acknowledged. First, high heterogeneity persisted despite subgroup analyses. Potential unaccounted sources include individualized TCM diagnostic variation (e.g., differences in herbal composition, decoction duration) and geo-ecological influences such as climate and regional humidity. Second, the most critical limitation was the universal absence of reported allocation concealment and inadequate or absent blinding of participants, personnel, and outcome assessors in all 39 included RCTs. This resulted in “high risk” judgments in key bias domains, introducing substantial potential for performance and detection bias that may have inflated effect estimates and undermined evidence certainty. Third, the lack of placebo-controlled trials precludes definitive attribution of therapeutic effects specifically to YPFS. Fourth, recurrence rates—a key measure of long-term efficacy—were insufficiently reported, only four studies provided relevant data, limiting statistical power and increasing the risk of false-negative results. Fifth, confounding factors such as seasonal allergen levels, air pollution, genetic variation, comorbidities, and other patient-specific characteristics were not systematically assessed in the included studies. This gap restricts our ability to generalize findings and may mask effect modifiers relevant to clinical practice. Sixth, the mechanistic evidence remains incomplete. While our meta-analysis focused on classical immune-inflammatory pathways, YPFS may also act through the regulation of additional immune cell types, signaling cascades, neurogenic inflammation, or epithelial barrier integrity. Sparse and inconsistent reporting prevented the incorporation of these factors into pooled analyses. Seventh, the included studies were exclusively from Chinese databases, lacking cross-ethnic or multi-regional validation, which limits external generalizability. Eighth, none of the trials were pre-registered on platforms such as PROSPERO, and selective outcome reporting—particularly of positive findings—may have exaggerated perceived efficacy. Finally, the safety profile of YPFS remains inadequately characterized. Adverse events were not systematically monitored in many studies, and when reported, they primarily covered subjective symptoms such as skin reactions or gastrointestinal discomfort without objective safety measures (e.g., liver or kidney function tests). This incomplete reporting may introduce bias and prevents a definitive safety assessment.

### 4.4 Recommendations for future TCM clinical studies

Based on the findings and limitations of this study, several recommendations are proposed for future clinical research on TCM. First, strengthen standardization by unifying the sourcing, processing, and decoction of Chinese herbal medicines, clarifying criteria for TCM pattern differentiation, and incorporating geographic and ecological factors as covariates in multicenter trials to reduce heterogeneity. Second, adhere to internationally recognized clinical research guidelines such as the CONSORT statement, ensuring rigorous study design with scientifically implemented randomization, blinded allocation, and allocation concealment. Use computer-generated random sequences and independent third-party allocation concealment, and conduct multicenter, large-sample studies to improve reliability and generalizability. Third, include placebo controls where possible to minimize the influence of psychological and other confounding factors, enabling accurate evaluation of the net efficacy of Chinese medicine. Fourth, establish standardized recurrence assessment criteria with clearly defined timepoints and metrics, and perform multicenter RCTs with large samples and extended follow-up (≥6 months) to better evaluate the long-term effects of YPFS on allergic rhinitis recurrence. Fifth, systematically document environmental exposures and patient characteristics, applying stratified analyses or multivariate regression to quantify their impact on outcomes, while expanding measured endpoints to include immune cell profiles, signaling pathways, neurogenic inflammation factors, and epithelial barrier function indicators. Adopt standardized data formats to improve understanding of YPFS mechanisms. Sixth, increase cross-racial and multi-geographical trials to capture data from diverse populations and enhance external validity. Seventh, standardize trial registration by requiring pre-registration and protocol submission for TCM studies, and strengthen monitoring of registered trials to avoid selective outcome reporting. Lastly, improve safety evaluation by following the CONSORT and STROBE guidelines, using standardized terminology for adverse event reporting, and combining patient-reported symptoms with regular laboratory monitoring of blood counts, liver and kidney function, and electrolytes for objective safety assessment and early detection of subclinical adverse effects. Given that YPFS is a herbal formula, future studies should also examine how raw material sourcing, processing techniques, and formulation modifications influence safety, and concurrently enhance post-marketing pharmacovigilance systems with robust data-sharing mechanisms.

## 5 Conclusion

Collectively, this study indicates that for AR management, YPFS combined with conventional pharmacotherapy may confer superior advantages over conventional pharmacotherapy alone in modulating immune homeostasis and suppressing inflammatory responses. Monotherapy with YPFS also demonstrates contributory immunomodulatory and anti-inflammatory effects. At the same time, YPFS can improve the overall response rate of clinical treatment, reduce the recurrence rate, and no major safety signals associated with YPFS have been identified. However, given the limited number of included studies, small sample sizes, poor methodological quality, and low quality of evidence, in particular, the included studies suffered from unreported or underreported key aspects such as blinding and allocation concealment, which greatly affected the quality of the studies and the reliability of the results. Therefore, the evidence from this study remains inconclusive and should be used with caution. When treating AR in the clinic, it is still necessary to consider the overall situation of the patient to develop a more appropriate and comprehensive treatment strategy. More high-quality, large-sample, multicenter, randomized, double-blind, and placebo-controlled trials are needed in the future, and these studies need to strictly follow better trial design criteria to obtain more reliable conclusions, so as to provide stronger evidence for the clinical application of YPFS for AR. In addition, the mechanism of action of YPFS for AR has not been fully elucidated, and in the future, the specific mechanism of action of YPFS to improve AR by regulating the intestinal flora can be further investigated from the perspectives of genes, transcription, and metabolism levels by utilizing multi-omics technology.

## Data Availability

The original contributions presented in the study are included in the article/Supplementary Material, further inquiries can be directed to the corresponding author.
