# Supplementary material for: The efficacy and safety of Yupingfengsan in the treatment of allergic rhinitis: a systematic review and meta-analysis
Source: Front Pharmacol. 2025 Sep 15;16:1628640. doi: 10.3389/fphar.2025.1628640 (PMC12477554; doi:10.3389/fphar.2025.1628640)
Supplement: Supplementary file 2 [file Supplementaryfile1.zip › Supplementary Material 4.0.docx]

supplementary material

catalog page

[1	Supplementary Material S1. Database and Search Strategies	2](#_Toc208494254)

[1.1	PubMed	2](#_Toc208494255)

[1.2	Embase	2](#_Toc208494256)

[1.3	Cochrane	2](#_Toc208494257)

[1.4	WOS	3](#_Toc208494258)

[1.5	CNKI	3](#_Toc208494259)

[1.6	Wan Fang	4](#_Toc208494260)

[1.7	VIP	4](#_Toc208494261)

[1.8	CBM	5](#_Toc208494262)

[1.9	ClinicalTrials.gov	6](#_Toc208494263)

[1.10	Chinese Clinical Trial Registry	6](#_Toc208494264)

[2	Supplementary Material S2. The procedure for preliminary screening of the literature	7](#_Toc208494265)

[3	Supplementary Material S3. Literature excluded after reading the full text and reasons	7](#_Toc208494266)

[3.1	The Interventions do not match	7](#_Toc208494267)

[3.2	No data on ending variables that meet the requirements	11](#_Toc208494268)

[3.3	Sample size <50	14](#_Toc208494269)

[3.4	Full text not available	14](#_Toc208494270)

[3.5	Mismatch of research subjects	14](#_Toc208494271)

[4	Supplementary Material S4. Characteristics of the included studies.	15](#_Toc208494272)

[5	Supplementary Material S5. The components of the original YPFS or modified YPFS	19](#_Toc208494273)

[6	Supplementary Material S6. Subgroup analysis	21](#_Toc208494274)

[6.1	Subgroup analysis of TNSS for YPFS combined with Conventional pharmacotherapy vs. Conventional pharmacotherapy	21](#_Toc208494275)

[6.2	Subgroup analysis of IgE for YPFS combined with Conventional pharmacotherapy vs. Conventional pharmacotherapy	21](#_Toc208494276)

[6.3	Subgroup analysis of IL-6 for YPFS combined with Conventional pharmacotherapy vs. Conventional pharmacotherapy	21](#_Toc208494277)

[7	Supplementary Material S7. Sensitivity analysis	22](#_Toc208494278)

[8	Supplementary Material S8. Sensitivity Analysis Based on Risk of Bias	24](#_Toc208494279)

[9	Supplementary Material S9. Egger’s test of TNSS, Overall effective rate, Adverse event rate and IL-6	25](#_Toc208494280)

[10	Supplementary Material S10. Clipping and Patching	25](#_Toc208494281)

[11	Supplementary Material S11. Adverse event	26](#_Toc208494282)

[12	Supplementary Material S12. Assessment of evidence quality for each outcome	29](#_Toc208494283)

# 1 Supplementary Material S1. Database and Search Strategies

## 1.1 PubMed

The retrieval of the PubMed database was conducted on December , 2024, and 4 records were retrieved.

## 1.2 Embase

The retrieval of the Embase database was conducted on December, 2024, and 6 records were retrieved.

## 1.3 Cochrane

The retrieval of the Cochrane Library was conducted on December, 2024, and 3 records were retrieved.

## 1.4 WOS

The retrieval of the WOS database was conducted on December, 2024, and 8 records were retrieved.

## 1.5 CNKI

The retrieval of the CNKI database was conducted on December, 2024, and a total of 287 records were retrieved.

## 1.6 Wan Fang

The retrieval of the Wan Fang database was conducted on December, 2024, and a total of 365 records were retrieved.

## 1.7 VIP

The retrieval of the VIP database was conducted on December, 2024, and a total of 272 records were retrieved.

## 1.8 CBM

The retrieval of the CBM database was conducted on December, 2024, and a total of 80 records were retrieved.

## 1.9 ClinicalTrials.gov

The retrieval of the ClinicalTrials.gov database was conducted on December, 2024, and 2 records were retrieved.

## 1.10 Chinese Clinical Trial Registry

The retrieval of the Chinese Clinical Trial Registry was conducted on December, 2024, and no records were retrieved.

# 2 Supplementary Material S2. The procedure for preliminary screening of the literature

# 3 Supplementary Material S3. Literature excluded after reading the full text and reasons

## 3.1 The Interventions do not match

‌[1] Bai, Y. and Liu, X.W.‌ (2023). Effects of Yupingfeng granules combined with fluticasone propionate nasal spray on ICAM-1 and Th17/Treg in patients with allergic rhinitis. Clin Med Res Pract, 8(23).
[2] Chen, H.K. and Jing, J.‌ (2016). Clinical efficacy of modified Yupingfeng San combined with loratadine tablets for allergic rhinitis. China Health Care Nutr, 26(14), 152.
[3] ‌Chen, J.B.‌ (2015). Clinical research of allergic rhinitis treated with integrative traditional Chinese and Western medicine. Henan Tradit Chin Med, 35(9).
[4] Chen, J., Wei, F.R., Nie, L.Y. and Qiao, F.Y.‌ (2013). Randomized, double-blind, parallel-controlled, multicenter clinical trial of Yupingfeng Capsules in the treatment of allergic rhinitis. J Pharm Res, 32(9).
[5] Chen, K., Luo, R., Li, D.X. and Ye, B.X.‌ (2004). Endoscopic radiofrequency thermocoagulation of anterior ethmoidal nerve combined with Yupingfeng granules for perennial allergic rhinitis. Chin J Otorhinolaryngol Integr Med, (04), 193-194. doi:10.16542/j.cnki.issn.1007-4856.2004.04.012
[6] Chen, L.L. and Zhao, J.‌ (2016). Efficacy of fluticasone propionate nasal spray (Flixonase) combined with Danxi Yupingfeng granules in children with allergic rhinitis. World Latest Med Inf, 16(38), 98+100.
[7] Chen, L. and Chen, X.W.‌ (2014). Analysis on therapeutic effect of cetirizine combined with Yupingfeng San in treating allergic rhinitis. World Chin Med, (7).
[8] Chen, S.J., Wang, W.H., Liu, B. and Zhao, X.Y.‌ (2015). Effects of azelastine spray combined with Yupingfeng San on serum IgE and peripheral eosinophils in allergic rhinitis. Mod J Integr Tradit Chin West Med, 24(36).
[9] Chen, X.M. and Meng, X.‌ (2022). Application of modified Yupingfeng decoction in lung-spleen qi deficiency-type allergic rhinitis. Mod Med Health Res, 6(16), 16-19.
[10] Deng, Y.D.‌ (2019). Effects of modified Yupingfeng powder on clinical symptoms of patients with seasonal allergic rhinitis. Clin J Chin Med, 11(12).
[11] Fan, J. and Fan, S.Q.‌ (2021). Clinical efficacy of Yupingfeng granules in allergic rhinitis. China Health Care Nutr, 31(21), 161-162.
[12] Fan, J.‌ (2016). Clinical observation of ebastine combined with Yupingfeng granules for allergic rhinitis. Hubei J Tradit Chin Med, 38(02), 37-38.
[13] Fan, Z.L.‌ (2021). Efficacy of Dermatophagoides farinae sublingual drops desensitization combined with Yupingfeng granules in allergic rhinitis. Chin Foreign Med Res, 19(25).
[14] Gan, H.Y.‌ (2018). Analysis of the curative effect of Yupingfeng Capsule combined with desloratadine citrate disodium tablets on allergic rhinitis. Chin Foreign Med Treat, 37(6).
[15] Gong, X.J.‌ (2024). Clinical study on Yupingfeng granules combined with sublingual desensitization with Dermatophagoides farinae drops for allergic rhinitis in children with lung-spleen qi deficiency. New Chin Med, 56(5).
[16] Gou, D.J.‌ (1991). Modified Yupingfeng San for 250 cases of allergic rhinitis. J Integr Tradit Chin West Med, (05), 288.
[17] Gu, L.L., Liu, G. and Zhu, L.‌ (2010a). Clinical observation of Sterimar nasal spray combined with Yupingfeng granules for allergic rhinitis. Paper presented at the 16th National Academic Conference of Otolaryngology Branch of CACMS, Beijing.
[18] Gu, L.L., Liu, G. and Zhu, L.‌ (2010b). Sterimar nasal spray combined with Yupingfeng granules for allergic rhinitis. Clin J Chin Med, 22(10), 871-872. doi:10.16448/j.cjtcm.2010.10.044
[19] Guo, J.L.‌ (2016). Clinical observation of modified Yupingfeng powder for seasonal allergic rhinitis. Sichuan J Tradit Chin Med, 34(6).
[20] Hou, S.X.‌ (2023). Effect of Yupingfeng granule combined with montelukast sodium and loratadine on allergic rhinitis. Chin Med Abstr Otorhinolaryngol, 38(5).
[21] Hou, T.P.‌ (2024). Influence of modified Yupingfeng decoction combined with western medicine on serum inflammatory factors and TCM syndromes in allergic rhinitis with lung-spleen qi deficiency. Chin J Pract Med, 51(18).
[22] Hua, L.‌ (2013). Improvement of Yupingfeng granules combined with loratadine on symptoms and immunity in allergic rhinitis. Mod J Integr Tradit Chin West Med, 22(12).
[23] Huang, J.J., Zhou, H., Huang, P. and Zhou, R.D.‌ (2022). Clinical effects of supplemented Yupingfeng decoction combined with western drug treatment on allergic rhinitis due to lung-spleen qi deficiency. Chin Tradit Pat Med, 44(2).
[24] Huang, R.J.‌ (2024). Efficacy of Yupingfeng granules in children with allergic rhinitis. Chin Sci J Database (Citation Ed) Med Health, (11).
[25] Jin, Y.X. and Li, X.L.‌ (2024). Effects of Yupingfeng granules combined with loratadine on symptoms and immunity in allergic rhinitis. Health Friend, (2), 164-166.
[26] Jing, S.L., Tang, X.R., Mo, L., Yang, Y., Wen, H., Hu, J.F. and Yu, X.‌ (2018). Efficacy of leukotriene receptor antagonists combined with Yupingfeng in children with allergic rhinitis. Mod Med Health, 34(A02).
[27] Li, M.Q.‌ (2015). Clinical observation of 117 cases of allergic rhinitis treated with integrated Chinese and Western medicine. Med Inf (Xi’an), (11).
[28] Li, R.F.‌ (2018). Efficacy of Yupingfeng granules combined with Flixonase in allergic rhinitis. Mod Diagn Treat, 29(2).
[29] Li, X.D.‌ (2016). Efficacy analysis of montelukast sodium chewable tablets combined with Yupingfeng capsules in children with allergic rhinitis. Paper presented at the 2016 Annual Conference of Zhejiang Medical Association Otolaryngology Branch, Huzhou, China.
[30] Li, X.Y.‌ (2013). Efficacy analysis of modified Yupingfeng San combined with cetirizine for 50 cases of allergic rhinitis. Jilin Med J, 34(3).
[31] Liu, C.J.‌ (2023). Clinical efficacy of modified Yupingfeng decoction in lung-spleen qi deficiency-type allergic rhinitis. Health Friend, (16), 150-151, 154. doi:10.3969/j.issn.1002-8714.2023.16.057
[32] Liu, F.‌ (2012). Efficacy observation of Yupingfeng granules in 70 cases of allergic rhinitis. Shandong Med J, 52(9), 87-88. doi:10.3969/j.issn.1002-266X.2012.09.037
[33] Liu, F.F. and Lu, M.Z.‌ (2013). Modified Yupingfeng San combined with triamcinolone acetonide local injection for 120 cases of allergic rhinitis. J Gansu Coll Tradit Chin Med, 30(4).
[34] Liu, L.P.‌ (2014). Sanhuang Yupingfeng ointment acupoint application in 50 cases of pediatric allergic rhinitis. J Tradit Chin Med Res, 27(2).
[35] Liu, X.Y., Ouyang, S.L. and Chu, Y.M.‌ (2014). Clinical effect of sublingual immunotherapy with Dermatophagoides farinae combined with Yupingfeng granule in allergic rhinitis. Chin J Otorhinolaryngol Integr Med, 22(6).
[36] Lu, L.‌ (2010a). Integrated traditional Chinese and Western medicine for 28 cases of allergic rhinitis. Henan Tradit Chin Med, 30(10).
[37] Lu, L.‌ (2010b). Clinical study on integrated traditional Chinese and Western medicine for allergic rhinitis. Paper presented at the Proceedings of the 11th National Academic Conference on New Technologies, Achievements, and Experiences in Traditional Chinese Medicine, Beihai, China.
[38] Ma, P., Feng, J. and Chen, B.L.‌ (2017). Yupingfeng granule combined with citric acid and desloratadine citrate disodium tablets in allergic rhinitis and its effects on immune function. Int J Tradit Chin Med, 39(10).
[39] Miao, K. and Zhang, J.C.‌ (2013). Comparative effect of Yupingfeng powder and Western medicine in the treatment of allergic rhinitis. Med Recapitul, 19(14).
[40] Ni, Z.‌ (2011). Efficacy observation of integrated Chinese and Western medicine in 75 cases of allergic rhinitis. Chin Med Front, 6(11).
[41] Peng, G.‌ (2014). Clinical observation of combined traditional Chinese and Western medicine for allergic rhinitis. J Clin Rational Drug Use, 7(1).
[42] Qin, Y., Ye, B.X., Liu, Y.Z. and Deng, Y.‌ (2017). Sublingual Dermatophagoides farinae drops combined with modified Yupingfeng decoction in allergic rhinitis. Hebei Med J, 23(2).
[43] Shi, J.K., Liu, G. and Wu, F.H.‌ (2019). Clinical observation of 90 cases of pediatric allergic rhinitis treated with sublingual immunotherapy (Dermatophagoides farinae) combined with Yupingfeng granule. Chin J Otorhinolaryngol Integr Med, 27(1).
[44] Su, Y.‌ (2009). Integrated traditional Chinese and Western medicine for 55 cases of allergic rhinitis. Chin J Ethnomed Ethnopharm, (15).
[45] Tian, W.R.‌ (1997). Integrated traditional Chinese and Western medicine for 160 cases of allergic rhinitis. Tianjin J Tradit Chin Med, 14(4).
[46] Tian, Y. and Li, X.‌ (2016). Jade screen particles combined therapy with loratadine tablets in patients with allergic rhinitis. Jilin J Tradit Chin Med, (8).
[47] Wang, B., Zhong, Y.F. and Hong, C.G.‌ (2015). Yupingfeng Granule combined with budesonide nasal spray in the treatment of allergic rhinitis for 45 cases. Chin Med Mod Distance Educ China, (23).
[48] Wang, F.F. and Wang, Y.‌ (2011). Analysis on the therapeutic effect of Yupingfeng decoction in the treatment of allergic coryza. J Med Coll PAPF, 20(10).
[49] Wang, H.Y. and Ci, J.‌ (2014). Efficacy of Yupingfeng particles combined with loratadine in treatment of allergic rhinitis of 50 cases. Chin J Mod Dr, 52(14).
[50] Wang, J.‌ (2009). Budesonide nasal spray combined with Yupingfeng granules for perennial allergic rhinitis. Jiangxi Med J, 44(9).
[51] Wang, K.C.‌ (2018). Clinical efficacy of modified Yupingfeng San in adjunctive treatment in children with allergic rhinitis and its effect on their immune function. Pediatr J Tradit Chin Med, (6).
[52] Wang, S.J.‌ (2010). Yupingfeng granules combined with budesonide nasal spray for 63 cases of allergic rhinitis. Chin Med Mod Distance Educ China, (21).
[53] Wang, T.‌ (2018). Modified Yupingfeng San combined with azelastine nasal spray for allergic rhinitis. J Pract Tradit Chin Med, 34(7).
[54] Wang, Y.‌ (2015). Clinical efficacy of Yupingfeng granules combined with loratadine tablets in allergic rhinitis. J Clin Rational Drug Use, 8(24).
[55] Wu, F.H., Gu, L.L. and Liu, G.‌ (2013). Clinical study of Yupingfeng granule nasal irrigation for allergic rhinitis with lung deficiency-cold syndrome. Clin J Tradit Chin Med, (1).
[56] Xu, H.X.‌ (2006). Modified Yupingfeng San for 30 cases of nasal obstruction. New Chin Med, 38(5).
[57] Xu, S.S., Chen, Z.Q. and Huang, Z.A.‌ (2018). Clinical observation of 41 cases of allergic rhinitis treated with Dermatophagoides farinae drops combined with modified Yupingfeng decoction. Chin J Ethnomed Ethnopharm, 27(2).
[58] Yang, L.X., Zhao, W.X. and Zhou, N.‌ (2013). Curative effect of unite Yupingfeng in allergic rhinitis patients. J Med Theory Pract, 26(20).
[59] Ye, M.H.‌ (2023). Clinical effect of modified Yupingfeng Powder combined with inferior turbinate injection of triamcinolone acetonide in adult lung qi deficiency-cold type allergic rhinitis. Chin Contemp Med, 30(27).
[60] Yu, D.Y.‌ (2016). Observation of the effect of modified Yupingfeng Powder in treating 30 cases of pediatric allergic rhinitis. Pediatr J Tradit Chin Med, 12(1).
[61] Zhang, B.G., Liang, J.H. and Yang, S.P.‌ (2017). Effects of desloratadine combined with Yupingfeng granules on peripheral blood CD34+ cells and eosinophils in pediatric allergic rhinitis. Paper presented at the Proceedings of the 2017 National Academic Conference of the Pediatric Education Research Committee of the National Higher Education Society of Traditional Chinese Medicine, Zhengzhou, China.
[62] Zhao, Z.X.‌ (2017). Clinical efficacy analysis of Yupingfeng granules combined with loratadine in allergic rhinitis. Health Guide, (52), 29. doi:10.3969/j.issn.1006-6845.2017.52.029
[63] Zheng, Y.‌ (2022). Efficacy of Yupingfeng granules combined with desloratadine citrate tablets in allergic rhinitis. Chin Sci J Database (Abstract Ed) Med Health, (12).

## 3.2 No data on ending variables that meet the requirements

[1] Chan, R.Y. and Chien, W.T.‌ (2014). The effects of two Chinese herbal medicinal formulae vs. placebo controls for treatment of allergic rhinitis: a randomised controlled trial. Trials, 15, 261. doi:10.1186/1745-6215-15-261.
[2] Bateer, S.Y. and Qi, J.‌ (2013). Clinical observation of modified Yupingfeng San in the treatment of allergic rhinitis. North Pharm J, 10(7), 30-31.
[3] Bao, S.E.‌ (2011). Clinical observation of loratadine combined with Yupingfeng granules for allergic rhinitis. J Pract Tradit Chin Med, 27(4).
[4] Cao, J.‌ (2019). Efficacy of Yupingfeng granules combined with loratadine tablets in allergic rhinitis. J Med Aesthet Cosmetol, 28(1), 83.
[5] Chen, T.R.‌ (2013). Clinical observation of integrated Chinese and Western medicine for allergic rhinitis. China Med Guide, 11(17).
[6] Deng, L.G.‌ (2019). Clinical efficacy of Yupingfeng powder in the treatment of allergic rhinitis in children and its effect on immune function. Chin Foreign Med Treat, 38(11).
[7] Dong, H.‌ (2012). Modified Yupingfeng San combined with cetirizine for 32 cases of allergic rhinitis. Mod J Integr Tradit Chin West Med, 21(9).
[8] Fan, R.M.‌ (2013). Yupingfeng granules combined with desloratadine for 36 cases of pediatric allergic rhinitis. Chin Med Mod Distance Educ China, (11).
[9] Guo, H.L., Liu, J.J. and Zhao, Y.L.‌ (2015). An effective analysis of treating allergic rhinitis with the Yupingfeng granules. Clin J Chin Med, 7(35).
[10] Guo, J.L., Hong, Z.S., Hong, H.Y. and Liao, W.‌ (2016). Evaluation of curative effect by Yupingfeng granules combined with budesonide nasal spray in the treatment of moderate-severe persistent allergic rhinitis. Chin Pract Med, 11(32).
[11] Guo, X. and Chen, D.S.‌ (2014). Fluticasone nasal spray combined with oral Yupingfeng capsule in the treatment of allergic rhinitis. Chin J Otorhinolaryngol Integr Med, 22(4).
[12] Han, L., Han, S.W. and Li, Q.‌ (2014). Clinical analysis of Yupingfeng San modified differential treatment of allergic rhinitis. Clin Med Eng, 21(7).
[13] Huang, J.‌ (2014). Clinical analysis of integrated Chinese and Western medicine for allergic rhinitis. For All Health (Acad Ed), 8(5), 17-18.
[14] Huang, P.‌ (2021). Efficacy of Yupingfeng granules combined with loratadine capsules in allergic rhinitis. Chin Sci J Database (Citation Ed) Med Health, (3).
[15] Li, H., Jin, S., Liu, L. and Guo, Y.F.‌ (2014). Yupingfeng granules combined with loratadine for allergic rhinitis. Hubei J Tradit Chin Med, 36(9), 38.
[16] Li, J.X.‌ (2017). Evaluation of Yupingfeng granules in the treatment of allergic rhinitis. Inner Mongolia J Tradit Chin Med, 36(4).
[17] Li, J.N. and Li, L.‌ (2010). Yupingfeng San combined with ablation for 130 cases of allergic rhinitis. Chin Med Mod Distance Educ China, 8(2).
[18] Li, W.M.‌ (2012). Integrated Chinese and Western medicine for 35 cases of allergic rhinitis. Chin Med Mod Distance Educ China, 10(15).
[19] Li, X.Y., Dong, J.P., Zeng, Y.J. and He, W.Y.‌ (2010). Clinical observation of 128 cases of pediatric allergic rhinitis. Chin Contemp Med, 17(35), 182-183.
[20] Li, Y.H.‌ (2012). Modified Yupingfeng San combined with mizolastine tablets for 60 cases of allergic rhinitis. Mod Med Health, 28(13).
[21] Lin, S.‌ (2013). Clinical observation of integrated Chinese and Western medicine for 60 cases of pediatric allergic rhinitis. Fujian J Tradit Chin Med, (2).
[22] Liu, W.‌ (2018). Effects of Yupingfeng granules combined with loratadine tablets on symptoms and immune function in allergic rhinitis. Chin J Mod Drug Appl, 12(10).
[23] Liu, Y.Z., Qin, Y. and Ye, B.X.‌ (2016). Efficacy of Yupingfeng San combined with mometasone furoate in allergic rhinitis. J Taishan Med Coll, (7).
[24] Liu, Z.H.‌ (2023). The effect of montelukast sodium combined with Yupingfeng granules in the treatment of children with allergic rhinitis and its influence on children's symptoms. Chin Med Abstr Otorhinolaryngol, 38(1).
[25] Lu, Y.Q.‌ (2014). Clinical observation of Yupingfeng granule in the adjuvant treatment of persistent allergic rhinitis. J Clin Exp Med, 13(7).
[26] Luo, H.X., Chen, K. and Li, D.X.‌ (2010). Rhinocort combined with Yupingfeng granules for perennial allergic rhinitis. Paper presented at the Proceedings of the 16th National Academic Conference of Otolaryngology Branch of CACMS, Beijing, China.
[27] Luo, Y.M. and Yi, W.M.‌ (2015). Clinical effects of Yupingfeng granule in the treatment of allergic rhinitis. Jilin Med J, 36(10).

‌[28] Luo, H.W.‌ (2012). Modified Yupingfeng San for 60 cases of spleen-lung deficiency-cold type allergic rhinitis. J Tradit Chin Med Res, 25(5).

[29] Pang, Z.Y.‌ (2011). Modified Yupingfeng San for 25 cases of allergic rhinitis. Henan Tradit Chin Med, 31(8), 933.

[30] Qiu, Q.L.‌ (2019). Clinical effect analysis of Yupingfeng granules combined with triamcinolone acetonide nasal spray in allergic rhinitis. North Pharm J, 16(9).

[31] Ru, X.P., Zeng, Y.Q. and Yan, S.‌ (2024). Effect of Yupingfeng decoction combined with budesonide nasal irrigation on patients with allergic rhinitis (syndrome of qi deficiency of lung and spleen) and its influence on inflammatory factors and immune function. Chin Med Innov, 21(31).

[32] Shi, H.Y., Zhuang, Y. and Wang, X.Y.‌ (2014). Effect of Yupingfeng drop pill in treatment of allergic rhinitis. China J Chin Mater Med, 39(12).

[33] Song, J.‌ (2018). Efficacy observation of Yupingfeng granules and desloratadine in allergic rhinitis. Electron J Clin Med Lit, 5(66), 136+138. doi:10.16281/j.cnki.jocml.2018.66.077

[34] Wang, X.D.‌ (2014). Clinical observation of modified Yupingfeng San for lung-spleen qi deficiency-type allergic rhinitis. J Med Aesthet Cosmetol (Mid-Issue), (9), 258.

[35] Wang, Y.J.‌ (2019). Analysis of the effect of Yupingfeng granule combined with montelukast in the treatment of allergic rhinitis in children. Liaoning Med J, (2).

[36] Wu, J.T. and Bai, Y.‌ (2021). Clinical efficacy of modified Yupingfeng San in differentiated treatment of allergic rhinitis. Inner Mongolia J Tradit Chin Med, 40(5).

[37] Xie, O.L.‌ (2011). Clinical observation of modified Yupingfeng San for 62 cases of allergic rhinitis. J Changchun Univ Tradit Chin Med, 27(5).

[38] Xie, X.B.‌ (2015). Clinical observation of Yupingfeng granules combined with budesonide nasal spray in allergic rhinitis. Inner Mongolia J Tradit Chin Med, 34(2).

[39] Xiong, C.Y.‌ (2019). Clinical study on the treatment of allergic rhinitis with Yupingfeng granule and desloratadine citrate disodium tablets. Med Front, 9(6).

[40] Xu, L., Zhang, X., Duan, B.Y. and Chen, Q.‌ (2020). Effect analysis of Yupingfeng powder combined with levocetirizine in the treatment of allergic rhinitis patients. Contemp Med, 26(18).

[41] Xu, S.F.‌ (2014). Efficacy observation of Yupingfeng granules combined with loratadine syrup in pediatric allergic rhinitis. Contemp Med Forum, (19).

[42] Yan, X.L.‌ (2011). Allergic rhinitis treated with modified Yupingfeng San in 115 cases. J Beijing Univ Tradit Chin Med, 34(5).

[43] Ye, B.X., Liu, Y.Z. and Qin, Y.‌ (2015). Clinical efficacy analysis of modified Yupingfeng San in differentiated treatment of allergic rhinitis. Inner Mongolia J Tradit Chin Med, 34(8).

[44] Ye, Q., Zeng, Y.H., Luo, Y.F., Wang, Y.W. and Tian, H.‌ (2014). Clinical observation of Yupingfeng granules combined with montelukast sodium in pediatric allergic rhinitis. Med Inf (Early Issue), (22).

[45] Zhang, G.Y., Li, J., Zhang, S., Su, T., Liu, L.P. and Qi, Y.Y.‌ (2023). Modified Yupingfeng decoction in treatment of allergic rhinitis with lung and spleen deficiency syndrome. Chin J Tradit Med, 38(12).

[46] Zhang, L.‌ (2019). Efficacy observation of Yupingfeng granules combined with azelastine hydrochloride nasal spray in allergic rhinitis. Contemp Med Forum, 17(7).

[47] Zhang, Q.‌ (2020). Clinical evaluation of Yupingfeng granule combined with triamcinolone acetonide nasal spray in the treatment of allergic rhinitis. Chin Community Dr, 36(2).

[48] Zhang, Y.‌ (2012). Efficacy observation of mometasone furoate combined with Yupingfeng granules in 55 cases of pediatric allergic rhinitis. Med Inf (Early Issue), 25(6).

[49] Zhao, L.H.‌ (2016). Clinical observation of Yupingfeng granules combined with mizolastine in allergic rhinitis. Chin J Mod Drug Appl, 10(24).

[50] Zhu, Y.L.‌ (2020). Efficacy observation of Yupingfeng San combined with budesonide nasal spray in allergic rhinitis. Health Guide, (5), 67.

## 3.3 Sample size <50

[1] Bai, H.P.‌ (2019). Efficacy observation of montelukast combined with Yupingfeng granules in the treatment of pediatric allergic rhinitis. Smart Health, 5(21), 139-140. doi:10.19335/j.cnki.2096-1219.2019.21.068.

[2] Yang, H.‌ (2010). Clinical observation of Yupingfeng granules in the prevention and treatment of 25 cases of pediatric allergic rhinitis. Pediatr J Tradit Chin Med, 6(2).

## 3.4 Full text not available

[1]	Fan, Y.Z., Zhang, X.C. and Zhao, Z.‌ (2016). Integrated Chinese and Western medicine for allergic rhinitis in flight personnel. Paper presented at the 8th National Conference on Integrated Chinese & Western Medicine for Allergic Diseases & 1st Shenzhen Conference on Integrated Chinese & Western Medicine for Allergic Diseases & 10th Shenzhen Respiratory Forum, Shenzhen, China.

## 3.5 Mismatch of research subjects

[1] Rao, C.L.‌ (2014). Clinical efficacy of montelukast combined with Yupingfeng granules in the treatment of 48 cases of mild persistent allergic rhinitis. J Guangzhou Med Univ, (3).

# 4 Supplementary Material S4. Characteristics of the included studies.

| Author/  Year | Sample size | Gender  (M/F) | Age  (years) | Course of disease | Dura-tion | Intervention | Outcom index | TCM pattern |
| --- | --- | --- | --- | --- | --- | --- | --- | --- |
| Chen  2017 | 170/ 150 | E: 100/70  C: 88/62 | E: 8.4±0.4  C: 8.3±0.3 | E: 3.9±0.2years  C: 3.8±0.1 years | 1month | E: Montelukast Sodium Tablets, 4mg qd + Yupingfeng Granules, 5g tid  C: Montelukast Sodium Tablets, 4mg qd | ③ | Mixed/Undifferentiated |
| Chen  2023 | 36/36 | E: 20/16  C: 21/15 | E: 8.14±1.29  C: 8.28±1.32 | E: 1.86±0.37 years  C: 1.95±0.42 years | 1month | E: Montelukast Sodium Tablets, 5mg qd + Yupingfeng Granules, 5g tid  C: Montelukast Sodium Tablets 5mg qd | ①④⑧ | Mixed/Undifferentiated |
| Cui, et al 2019 | 28/28 | E: 16/12  C: 18/10 | E: 39.85 ±5.07  C: 39.85 ±5.07 | NR | 2weeks | E: Fluorine propioNRte NRsal spray, qd + Yupingfeng Granules, 15g tid  C: Fluorine propioNRte NRsal spray, qd | ③④ | Lung Qi Deficiency Pattern |
| Deng, et al 2019 | 63/63 | E: 36/27  C: 35/28 | E: 43.7±4.9  C: 43.3±4.8 | E: 16.3±3.1 years  C: 16.4±3.4 years | 2weeks | E: Desloratadine, 1 tablet qd + Yupingfeng Granules, 5g tid  C: Desloratadine, 1 tablet qd | ③④ | Mixed/Undifferentiated |
| Dong  2021 | 45/45 | E: 21/24  C: 21/24 | E: 36.98±2.61  C: 35.23±2.34 | E: 7.53±1.77 years  C: 6.78±1.71 years | 4weeks | E: Budesonide Spray 200μg /spray, bid + Yupingfeng Granules, 5g tid  C: Budesonide Spray 200μg /spray, bid | ③⑦ | Mixed/Undifferentiated |
| Du, et al 2017 | 54/54 | E: 30/24  C: 32/22 | E: 7.64±2.15  C: 7.58±2.23 | E: 36.83±8.25 months  C: 36.76±8.32 months | 4weeks | E: Loratadine, 10mg qd + Yupingfeng Granules, tid  C: Loratadine, 10mg qd | ③ | Lung Qi Deficiency Pattern |
| Fang, et al 2014 | 118/102 | E: 53/65  C: 43/59 | E: 35.18±13.72  C: 36.03±12.85 | E: 3.26±1.27 years  C: 3.17±1.12 years | 1month | E: Yupingfeng capsule 0.5g /capsule, 1g tid  C: Cetirizine Hydrochloride Dispersible Tablets, 10mg qd + Budesonide Spray 200μg /spray, bid | ②③⑦ | Mixed/Undifferentiated |
| Fang, et al 2017 | 30/30 | E: 20/10  C: 20/10 | E: 7.45±0.82  C: 7.82±0.98 | E: 2.78± 0.35 years  C: 2.78± 0.35 years | 1month | E: Montelukast Sodium Tablets, 5mg qd + Yupingfeng Granules, 5g tid  C: Montelukast Sodium Tablets, 5mg qd | ②③④⑧ | Mixed/Undifferentiated |
| Guan  2013 | 27/26 | NR | E: 35.79±12.16  C: 35.79±12.16 | NR | 14d days | E: Budesonide Spray 200μg /spray, 64μg /side bid + Yupingfeng Granules, 5g tid  C: Budesonide Spray 200μg /spray, 64μg /per bid | ③④ | Mixed/Undifferentiated |
| Hong, et al 2023 | 65/65 | E: 35/30  C: 38/27 | E: 46.29±8.35  C: 45.47±8.21 | NR | 8weeks | E: Yupingfeng Granules, 5g tid  C: Mometasone Furoate NRsal Spray 50μg/ spray, 2 sprays per nostril, qd | ②④⑧ | Lung-Spleen Qi Deficiency Pattern |
| Huang 2024 | 40/40 | E: 24/16  C: 21/19 | E: 32.98±9.04  C: 33.05±8.98 | NR | 14 days | E: Budesonide Spray 200μg /spray, 64μg /per bid + Yupingfeng Granules, 5g tid  C: Budesonide Spray, 200μg /spray, 64μg /per bid | ①②③⑦ | Mixed/Undifferentiated |
| Jiang  2024 | 55/55 | E: 28/27  C: 26/29 | E: 35.78±6.18  C: 37.76±5.25 | E: 3.88±1.47 years  C: 5.23±2.32 years | 4weeks | E: Loratadine, 10mg qd + Mometasone Furoate NRsal Spray, qd + Yupingfeng Granules, 5g tid  C: Loratadine, 10mg qd + Mometasone Furoate NRsal Spray, qd | ①②③④⑦ | Lung-Spleen Qi Deficiency Pattern |
| Jing, et al 2019 | 60/60 | E: 35/25  C: 38/22 | E: 6.5±1.2  C: 6.7±1.5 | E: 35.2±8.6 months  C: 32.6±14.3 months | 1month | E: Loratadine, 5-10mg qd + Yupingfeng Granules, qd  C: Loratadine, 5-10mg qd | ③ | Lung Qi Deficiency Pattern |
| Kong, et al 2016 | 80/80 | E: 47/33  C: 44/36 | E: 40.63±6.74  C: 39.45±7.47 | E: 5.63±3.28 years  C: 5.49±3.22 years | 4weeks | E: Azelastine Hydrochloride NRsal Spray, 2 sprays per nostril bid + Yupingfeng Granules, 5g tid  C: Azelastine Hydrochloride NRsal Spray, 2 sprays per nostril | ①②③④⑦ | Mixed/Undifferentiated |
| Li, et al 2019 | 63/63 | E: 36/27  C: 35/28 | E: 43.7±4.9  C: 43.3±4.8 | E: 16.3±3.1 years  C: 16.4±3.4 years | 2weekss | E: Desloratadine, 1 tablet qd + Yupingfeng Granules, 5g tid  C: Desloratadine, 1 tablet qd | ③④ | Lung-Spleen Qi Deficiency Pattern |
| Li, et al 2022 | 30/30 | E: 20/10  C: 20/10 | E: 7.45±0.82  C: 7.82±0.98 | E: 2.78± 0.35 years  C: 2.78± 0.35 years | 2months | E: Loratadine, 10mg qd + Mometasone Furoate NRsal Spray 50μg/spray, 1 spray per nostril, qd + Yupingfeng Granules, 5g tid  C: Loratadine, 10mg qd + Mometasone Furoate NRsal Spray | ①③④⑧ | Lung-Spleen Qi Deficiency Pattern |
| Li  2023 | 41/41 | E: 18/23  C: 19/22 | E: 43.27±3.95  C: 43.19±4.27 | E: 6.27±1.45 years  C: 6.31±1.52 years | 14day | E: Yupingfeng Granules, 5g tid  C: Loratadine, 5mg qd | ③ | Mixed/Undifferentiated |
| Liao  2019 | 30/30 | E: 13/17  C: 14/16 | E: 44.2±5.2  C: 43.2±2.7 | E: 8.2±3.7 years  C: 7.6±3.7 years | 3months | E: Fluorine propioNRte NRsal spray, 2 spray per nostril, bid + Yupingfeng Granules, 15g tid  C: Fluorine propioNRte NRsal spray, 2 spray per nostril, bid | ① | Lung Qi Deficiency Pattern |
| Lin, et al 2024 | 48/48 | E: 23/25  C: 20/28 | E: 32.14±10.58  C: 31.57±10.62 | E: 2.10±0.42 years  C: 2.14±0.51 years | 1month | E: Desloratadine, 10mg qd + Montelukast Sodium Tablets, 10mg qd + Mometasone Furoate NRsal Spray 50μg/spray, 2 spray per nostril, qd + Yupingfeng Granules, 5g tid  C: Desloratadine, 10mg qd + Montelukast Sodium Tablets, 10mg qd + Mometasone Furoate NRsal Spray 50μg/spray, 2 spray per nostril, qd | ⑥ | Lung Qi Deficiency Pattern |
| Lv  2024 | 48/48 | E: 22/26  C: 25/23 | E: 6.5±1.5  C: 6.3±1.2 | E: 25.5± 3.6 months  C: 25.1±3.4 months | 1month | E: Montelukast Sodium Tablets, 5/10mg qd + Yupingfeng Granules, 2.5/5g tid  C: Montelukast Sodium Tablets, 5/10mg qd | ③ | Mixed/Undifferentiated |
| Ma  2017 | 37/37 | E: 21/16  C: 20/17 | E: 38.6±6.9  C: 37.9±6.5 | E: 5.1±0.3 years  C: 5.3±0.6 years | 4weeks | E: Azelastine NRsal Spray, 2 sprays per nostril, bid + Yupingfeng Granules, 5g tid  C: Azelastine NRsal Spray, 2 sprays per nostril, bid | ②⑤⑦⑧ | Lung Qi Deficiency Pattern |
| Pan  2024 | 31/31 | E: 17/14  C: 16/15 | E: 35.30±4.82  C: 35.26±4.31 | E: 3.02±0.65 years  C: 2.94±0.64 years | 2months | E: Azelastine Hydrochloride NRsal Spray, 2 sprays per nostril, bid + Yupingfeng Granules, 5g tid  C: Azelastine Hydrochloride NRsal Spray, 2 sprays per nostril | ①④⑥⑧ | Lung-Spleen Qi Deficiency Pattern |
| Qiu  2017 | 50/50 | E: 20/10  C: 20/10 | E: 7.45±0.82  C: 7.82±0.98 | E: 2.78± 0.35 years  C: 2.78± 0.35 years | 2weeks | E: Loratadine, 10mg qd + Mometasone Furoate NRsal Spray 50μg/spray, 1 spray per nostril, qd + Yupingfeng Granules, 5g tid  C: Loratadine 10mg qd + Mometasone Furoate NRsal Spray | ③④ | Mixed/Undifferentiated |
| Qiao  2021 | 60/60 | E: 36/27  C: 35/28 | E: 43.7±4.9  C: 43.3±4.8 | E: 16.3±3.1 years  C: 16.4±3.4 years | 4weeks | E: Montelukast Sodium Tablets, 10mg qd + Mometasone Furoate NRsal Spray 50μg/spray, 2 spray per nostril, qd + Yupingfeng Granules, 5g tid  C: Montelukast Sodium Tablets, 10mg qd + Mometasone Furoate NRsal Spray 50μg/spray, 2 spray per nostril, qd | ③④ | Mixed/Undifferentiated |
| Wan, et al 2021 | 148/  148 | E: 70/74  C: 68/74 | E: 39±2.36  C: 38.6±4.66 | NR | 4weeks | E: Desloratadine Citrate Dispersible Tablets, 8.8mg qd + Yupingfeng Granules, 5g tid  C: Desloratadine Citrate Dispersible Tablets, 8.8mg qd | ②③④⑧ | Mixed/Undifferentiated |
| Wang , et al 2019 | 100/  100 | E: 64/36  C: 63/37 | E: 46.8  C: 47.8 | E: 5.1 months  C: 4.8 months | 2weeks | E: Triamcinolone Acetonide NRsal Spray, 2 sprays per nostril, qd + Yupingfeng Granules, 5g tid  C: Triamcinolone Acetonide NRsal Spray, 2 sprays per nostril, qd | ③④ | Mixed/Undifferentiated |
| Wang, et al 2021 | 100/  100 | E: 49/51  C: 52/48 | E: 32.2±2.1  C: 31.1±2.3 | E: 2.6±0.5 years  C: 2.3±0.4 years | 4weeks | E: Mometasone Furoate NRsal Spray 50μg/spray, 2 spray per nostril, qd + Yupingfeng Granules, 5g tid  C: Mometasone Furoate NRsal Spray 50μg/spray, 2 spray per nostril, qd | ③④ | Mixed/Undifferentiated |
| Wang, et al 2022 | 43/43 | E: 25/18  C: 24/19 | E: 31.17±1.49  C: 30.69±1.57 | E: 30.58±1.48 months  C: 31.17±1.25 months | NR | E: Montelukast Sodium Tablets, 10mg qd + Loratadine, 10mg qd + Yupingfeng Granules, 5g tid  C: Montelukast Sodium Tablets,10mg qd + Loratadine, 10mg qd | ③⑥ | Mixed/Undifferentiated |
| Wu, et al 2016 | 107/  106 | E: 62/45  C: 60/46 | E: 43.14±9.43  C: 41.84±5.36 | E: 9.83±4.69 years  C: 8.94±5.38 years | 14days | E: Setastine Hydrochloride Tablets, 1mg bid + Yupingfeng Granules, 5g tid  C: Setastine Hydrochloride Tablets, 1mg bid | ③④ | Mixed/Undifferentiated |
| Wu, et al 2018 | 36/36 | E: 20/16  C: 22/14 | E: 7.08±1.19  C: 7.25±1.22 | E: 2.56±0.96 years  C: 2.65±0.85 years | 4weeks | E: Loratadine, 5/10mg qd + Yupingfeng Granules, 5g tid  C: Loratadine, 5/10mg qd | ②③④⑤ | Mixed/Undifferentiated |
| Wang  2023 | 40/40 | E: 21/19  C: 22/18 | E: 38.26±5.56  C: 38.16±5.32 | E: 39.90±8.16 months  C: 38.16±5.32 months | 30days | E: Loratadine, 5mg qd + Budesonide NRsal Spray 64μg/spray, 1 spray per nostril, bid + Modified Yupingfeng Powder, bid  C: Loratadine, 5mg qd + Budesonide NRsal Spray 64μg/spray, 1 spray per nostril, bid | ①②③ | Lung Qi Deficiency Pattern |
| Yang  2021 | 40/40 | E: 24/16  C: 26/14 | E: 3.12±2.32  C: 3.23±2.32 | E: 18.53±6.65 months  C: 18.55±5.12 months | 2weeks | E: Montelukast Sodium Tablets, 2/4mg qd + Yupingfeng Granules, 5g tid  C: Montelukast Sodium Tablets, 2/4mg qd | ③④ | Mixed/Undifferentiated |
| Yang, et al 2018 | 63/63 | E: 33/30  C: 34/29 | E: 8.57±2.12  C: 8.63±2.07 | E: 1.81±0.44 years  C: 1.83±0.42 years | 4weeks | E: Montelukast Sodium Tablets, 4/5mg qd + Yupingfeng Granules, 5g tid  C: Montelukast Sodium Tablets, 4/5mg qd | ③④ | Mixed/Undifferentiated |
| Yu, et al 2015 | 36/36 | E: 19/17  C: 16/20 | E: 7.3±3.5  C: 7.4±3.6 | E: 4.8±2.5 years  C: 4.9±2.7 years | 30days | E: Montelukast Sodium Tablets, 4mg qd + Yupingfeng Granules, 5g tid  C: Montelukast Sodium Tablets, 4mg qd | ②③④ | Mixed/Undifferentiated |
| Zhang, et al 2019 | 45/45 | E: 25/20  C: 28/17 | E: 29±3.5  C: 30±4.0 | E: 4.1±0.2 years  C: 4.2±0.3 years | 4weeks | E: Loratadine, 10mg qd + Yupingfeng Granules, 5g tid  C: Loratadine, 10mg qd | ①③④ | Mixed/Undifferentiated |
| Zhang, et al 2023 | 81/81 | E: 31/23  C: 30/24 | E: 22.31±3.14  C: 22.38±3.23 | E: 34.71±1.04months  C: 34.64±1.05 months | 4weeks | E: Levocetirizine Hydrochloride, 10mg qd + Yupingfeng Granules, 5g tid  C: Levocetirizine Hydrochloride, 10mg qd | ③⑦⑧ | Mixed/Undifferentiated |
| Zhang 2023 | 54/54 | E: 20/10  C: 20/10 | E: 7.45±0.82  C: 7.82±0.98 | E: 2.78± 0.35 years  C: 2.78± 0.35 years | 4weeks | E: Montelukast Sodium Tablets, 10mg qd + Yupingfeng Powder  C: Montelukast Sodium Tablets, 10mg qd | ①③④ | Lung Qi Deficiency Pattern |
| Zheng, et al 2017 | 36/36 | E: 18/18  C: 20/16 | E: 42.7±12.3  C: 43.1±11.6 | E: 7.7±1.3 years  C: 8.1±1.5 years | 4weeks | E: Levocetirizine Hydrochloride Tablets, 5mg qd + Yupingfeng Granules, 5g tid  C: Levocetirizine Hydrochloride Tablets, 5mg qd | ①③④ | Mixed/Undifferentiated |
| Zhao  2020 | 75/75 | E: 40/35  C: 39/36 | E: 4.0±1.5  C: 5.0±0.5 | E: 7.8±1.1weeks  C: 7.9±1.2 weeks | 4weeks | E: Cetirizine Hydrochloride Syrup, 5ml qd + Montelukast Sodium Chewable Tablets, 5mg qd + Modified Yupingfeng Powder, bid  C: Cetirizine Hydrochloride Syrup, 5ml qd + Montelukast Sodium Chewable Tablets, 5mg qd | ①③ | Lung Qi Deficiency Pattern |

Abbreviations: F, female; M, male; NR, not reported; E, experimental group; C, control group; Outcome index: ①TNSS; ②IgE; ③Overall effectiveness rate; ④Adverse event rate; ⑤Th1/Th2; ⑥Treg/Th17; ⑦IL-4; ⑧IL-6; ⑨Relapse rate.

# 6 Supplementary Material S5. The components of the original YPFS or modified YPFS

| Study | Formula | Components | Dosage  form | Source of information |
| --- | --- | --- | --- | --- |
| Jing, et al 2019 | Original YPFS | astragali mongholici radix 30g, atractylodis macrocephalae rhizoma 10g, saposhnikoviae radix 10g. | powder | Reported in original study |
| Du, et al 2017 | Original YPFS | astragali mongholici radix 30g, atractylodis macrocephalae rhizoma 10g, saposhnikoviae radix 10g. | powder | Reported in original study |
| Wang 2023 | Modified YPFS | astragali mongholici radix 60g, atractylodis macrocephalae rhizoma 60g, saposhnikoviae radix 30g. For itching of eyes, throat, and nose, Add herba schizonepetae 10g and flos chamomilla 10g. For cough Add fritillariae cirrhosae bulbus 6g and mori folium 12g. | Decoction | Reported in original study |
| Zhang, et al 2023 | Modified YPFS | astragali mongholici radix 12g, atractylodis macrocephalae rhizoma 12g, saposhnikoviae radix 6g. For severe nasal itching and sneezing, Add herba schizonepetae、cicadae periostracum. For severe nasal congestion, Add Angelicae Dahuricae Radix and cicadae periostracum. For profuse clear nasal discharge, Add alpiniae oxyphyllae fructus、linderae radix. | Decoction | Reported in original study |
| Zhao 2020 | Modified YPFS | astragali mongholici radix 12g, atractylodis macrocephalae rhizoma 12g, saposhnikoviae radix 6g. For severe nasal itching, Add cicadae periostracum 6g and bombycis corpus caducum 9g. If accompanied by wind aversion, cold intoleranc, and profuse watery nasal discharge, Add cinnamomi cortex 6g、zingiberis rhizoma 6g、ziziphi oenopoliae radix. | Decoction | Reported in original study |
| Remaining studies | Original YPFS | astragali mongholici radix, atractylodis macrocephalae rhizoma, saposhnikoviae radix. | Commercial Chinese Polyherbal Preparation | Pharmacopoeia of the People's Republic of China（） |

# 8 Supplementary Material S6. Subgroup analysis

## 8.1 Subgroup analysis of TNSS for YPFS combined with Conventional pharmacotherapy vs. Conventional pharmacotherapy

## 8.2 Subgroup analysis of IgE for YPFS combined with Conventional pharmacotherapy vs. Conventional pharmacotherapy

## 8.3 Subgroup analysis of IL-6 for YPFS combined with Conventional pharmacotherapy vs. Conventional pharmacotherapy

# 9 Supplementary Material S7. Sensitivity analysis

(A)TNSS; (B)IgE; (C) IgE’; (D) Overall effective rate; (E) Overall effective rate’; (F) Adverse events rate; (G) Th1/Th2; (H) Treg/Th17; (I) IL-4; (J) IL-6; (K) Relapse rate.

# 11 Supplementary Material S8. Sensitivity Analysis Based on Risk of Bias

(A)TNSS; (B)IgE; (C) IL-4; (D) IL-6; (E) Treg/Th17; (F) Overall effective rate; (G) Adverse events rate.

# 12 Supplementary Material S9. Egger’s test of TNSS, Overall effective rate, Adverse event rate and IL-6

(A)TNSS; (B) Overall effective rate; (C) Adverse event rate; (D)IL-6

# 13 Supplementary Material S10. Clipping and Patching

(A)TNSS; (B) Overall effective rate

# 14 Supplementary Material S11. Adverse event

| Auther  Year | Groups | Digestive system | | | Nervous system | | | Skins  Reactive | | Organ toxicity | | Other | | | Incidence of adverse events | Severity | Clinical significance |
| --- | --- | --- | --- | --- | --- | --- | --- | --- | --- | --- | --- | --- | --- | --- | --- | --- | --- |
|  |  | Nausea and vomiting | Constipation | Gastrointestinal discomfort | Spin | Headaches | Drowsiness | Rash | Pruritic | Nasal dryness | Nosebleed | Fatigue | Thirst | Other |  |  |  |
| Chen 2023 | E |  |  |  |  |  |  |  |  | 1 |  |  | 1 |  | 5.56% | NR | Combining with YPFS can reduce medication side effects. |
|  | C |  |  |  |  |  | 1 |  |  | 1 |  |  | 2 |  | 11.11% |  |  |
| Cui, et al 2019 | E |  |  |  |  |  |  |  |  | 1 | 1 |  |  | 1 | 10.71% | NR | NR |
|  | C |  |  |  |  |  |  |  |  | 2 | 4 |  |  | 1 | 25% |  |  |
| Deng, et al 2019 | E | 1 |  |  |  |  |  |  |  |  |  |  |  |  | 1.59% | NR | NR |
|  | C | 1 |  |  | 2 |  |  |  |  |  |  |  | 1 |  | 6.35% |  |  |
| Fang, et al 2017 | E |  |  | 1 |  | 2 | 1 |  |  |  |  |  |  |  | 13.33% | NR | The combined therapy of Montelukast and YPFS demonstrates a higher safety profile. |
|  | C |  |  | 2 |  | 4 | 2 |  |  |  |  |  | 2 |  | 33.33% |  |  |
| Guan  2013 | E |  |  |  |  |  | 1 |  |  |  |  |  |  | 2 | 11.11% | Symptoms disappeared after conservative treatment. | The combination therapy of Budesonide and YPFS is safe and effective in treating allergic rhinitis. |
|  | C |  |  |  |  |  |  | 2 |  |  |  |  |  | 2 | 15.38% | NR |  |
| Hong, et al 2023 | E | NR | | | | | | | | | | | | | 9.23% | NR | YPFS can be used to treat allergic rhinitis without any significant adverse reactions. |
|  | C |  |  |  |  |  |  |  |  |  |  |  |  |  | 16.92% |  |  |
| Kong, et al 2016 | E |  |  |  | 1 |  | 1 |  |  |  |  |  |  |  | 2.5% | NR | NR |
|  | C |  |  |  | 2 | 2 | 1 |  |  |  |  |  |  | 2 | 8.75% |  |  |
| Li, et al 2019 | E | 1 |  |  |  |  |  |  |  |  |  |  |  |  | 1.59% | NR | The combination of YPFS and loratadine can reduce the incidence of adverse reactions. |
|  | C | 1 |  |  | 2 |  |  |  |  |  |  |  | 1 |  | 6.35% |  |  |
| Li, et al 2022 | E |  |  |  |  |  | 1 |  |  |  |  |  |  | 3 | 10% | NR | The difference in adverse reactions between YPFS and Western medicine is not statistically significant. |
|  | C |  |  | 1 |  |  | 1 |  |  |  |  |  |  | 1 | 13.33% |  |  |
| Lv  2024 | E | 1 |  |  |  | 1 | 1 |  |  |  |  |  | 1 |  | 8.33% | NR | NR |
|  | C | 2 |  |  |  | 1 | 2 |  |  |  |  |  | 3 |  | 16.67% |  |  |
| Pan  2024 | E |  |  | 1 |  | 2 |  |  |  |  |  |  | 1 |  | 12.9% | NR | The difference in adverse reactions between YPFS and Western medicine is not statistically significant. |
|  | C |  |  | 1 |  | 1 | 2 |  |  |  | 1 |  | 1 |  | 19.35% |  |  |
| Qiao  2021 | E | 1 |  |  |  |  |  |  |  |  |  |  |  |  | 1.67% | NR | The combination of YPFS and loratadine can reduce the incidence of adverse reactions. |
|  | C | 2 |  |  | 3 |  |  |  |  |  |  |  | 2 |  | 11.67% |  |  |
| Wan, et al 2021 | E |  |  |  |  |  |  |  |  |  |  |  |  |  | 12.5% | NR | NR |
|  | C |  |  |  |  |  |  |  |  |  |  |  |  |  | 26.06% |  |  |
| Wang, et al 2019 | E |  |  |  | 2 | 1 | 2 |  |  |  |  |  |  |  | 5% | NR | The difference in adverse reactions between YPFS and Western medicine is not statistically significant. |
|  | C |  |  |  | 3 | 2 | 1 |  |  |  |  |  |  |  | 6% |  |  |
| Wang, et al 2021 | E |  |  |  | 1 |  |  |  |  |  |  |  |  |  | 1% | NR | YPFS demonstrate safety. |
|  | C |  |  |  | 3 |  |  |  |  |  |  |  |  |  | 3% |  |  |
| Wu, et al 2016 | E |  |  |  | 1 |  | 1 |  |  |  |  |  |  |  | 1.87% | NR | NR |
|  | C |  |  |  | 2 | 2 | 1 |  |  |  |  |  |  | 1 | 5.66% |  |  |
| Wu, et al 2018 | E |  |  | 1 |  | 3 | 1 |  |  |  |  |  |  |  | 13.9% |  | ‌Combination of YPFS and Loratadine Can Reduce the Incidence of Adverse Reactions |
|  | C |  |  | 2 |  | 5 | 2 |  |  |  |  |  | 3 |  | 33.4% |  |  |
| Yang, et al 2018 | E | 1 | 1 |  |  |  | 1 |  |  |  |  |  |  |  | 6.35% | NR | NR |
|  | C | 2 |  |  |  |  | 1 |  |  |  |  |  | 1 |  | 4.76% |  |  |
| Yang  2021 | E |  |  | 1 |  |  | 1 |  |  |  |  |  | 2 |  | 7.5% | NR | YPFS have few adverse reactions. |
|  | C |  |  | 1 |  | 1 |  |  |  |  |  |  | 1 |  | 10% |  |  |
| Yu, et al 2015 | E |  |  | 2 |  | 1 | 1 | 1 |  |  |  |  | 2 |  | 19.4% | NR | YPFS combined with Singulair is safe and reliable |
|  | C |  |  | 8 |  | 6 |  | 2 |  |  |  |  | 3 |  | 61.1% |  |  |
| Zhang 2023 | E |  |  |  |  |  |  | 1 |  |  |  |  |  | 1 | 3.7% |  | YPFS combined with Montelukast Sodium can reduce the incidence of adverse reactions. |
|  | C | 1 |  |  |  | 1 |  | 2 |  |  |  |  |  | 3 | 14.81% |  |  |

Abbreviations: E, experimental group; C, control group; NR, not reported.

# 16 Supplementary Material S12. Assessment of evidence quality for each outcome

| **Quality assessment** | | | | | | | | | | **No of patients** | | | **Effect** | | | **Quality** | | **Importance** |
| --- | --- | --- | --- | --- | --- | --- | --- | --- | --- | --- | --- | --- | --- | --- | --- | --- | --- | --- |
| **No of studies** | **Design** | **Risk of bias** | **Inconsistency** | **Indirectness** | | **Imprecision** | | **Other considerations** | | **Treatment** | | **C** | **Relative (95% CI)** | | **Absolute** |  |  |  |
| **TNSS (Better indicated by lower values)** | | | | | | | | | | | | | | | | | | |
| 12 | randomised trials | serious^1^ | very serious^2^ | no serious indirectness | | no serious imprecision | | none | | 552 | | 552 | - | | SMD 3.94 lower  (4.92 to 2.96 lower) | ⊕OOO VERY LOW | | CRITICAL |
| **IgE (Better indicated by lower values)** | | | | | | | | | | | | | | | | | | |
| 8 | randomised trials | serious^1^ | very serious^2^ | no serious indirectness | | no serious imprecision | | none | | 462 | | 460 | - | | SMD 2.11 lower  (2.53 to 1.69 lower) | ⊕OOO VERY LOW | | CRITICAL |
| **Overall effectiveness rate** | | | | | | | | | | | | | | | | | | |
| 32 | randomised trials | serious^1^ | no serious inconsistency | no serious indirectness | | serious^2^ | | none | | 1850/1939  (95.4%) | | 1542/1915  (80.5%) | RR 1.18  (1.16 to 1.21) | | 145 more per 1000  (from 129 more to 169 more) | ⊕⊕OO LOW | | CRITICAL |
|  |  |  |  |  |  |  |  |  |  |  | | 80.7% |  |  | 145 more per 1000 (from 129 more to 169 more) |  |  |  |
| **Adverse event rate** | | | | | | | | | | | | | | | | | | |
| 20 | randomised trials | serious^1^ | no serious inconsistency | | no serious indirectness | | serious^2^ | none | 76/1180  (6.4%) | | 165/1169  (14.1%) | | RR 0.46 (0.36 to 0.59) | 76 fewer per 1000  (from 58 fewer to 90 fewer) | | | ⊕⊕OO LOW | CRITICAL |
|  |  |  |  |  |  |  |  |  |  | | 11.4% | |  | 62 fewer per 1000  (from 47 fewer to 73 fewer) | | |  |  |

| **Th1/Th2 (Better indicated by lower values)** | | | | | | | | | | | | | | | |
| --- | --- | --- | --- | --- | --- | --- | --- | --- | --- | --- | --- | --- | --- | --- | --- |
| 3 | randomised trials | serious^1^ | | serious^2^ | no serious indirectness | | serious^3^ | none | | 153 | 153 | - | SMD 1.28 higher  (0.78 to 1.78 higher) | ⊕OOO VERY LOW | IMPORTANT |
| **Treg/Th17 (Better indicated by lower values)** | | | | | | | | | | | | | | | |
| 3 | randomised trials | serious^1^ | | very serious^2^ | no serious indirectness | | serious^3^ | none | | 122 | 122 | - | SMD 1.84 higher  (1.06 to 2.62 higher) | ⊕OOO VERY LOW | IMPORTANT |
| **IL-4 (Better indicated by lower values)** | | | | | | | | | | | | | | | |
| 4 | randomised trials | serious^1^ | very serious^2^ | | no serious indirectness | serious^3^ | | none | | 217 | 217 | - | SMD 1.27 lower  (1.83 to 0.71 lower) | ⊕OOO VERY LOW | IMPORTANT |
| **IL-6 (Better indicated by lower values)** | | | | | | | | | | | | | | | |
| 10 | randomised trials | serious^1^ | very serious^2^ | | no serious indirectness | | no serious imprecision | | none | 564 | 562 | - | SMD 2.21 lower  (2.88 to 1.53 lower) | ⊕OOO VERY LOW | IMPORTANT |
| **Relapse rate** | | | | | | | | | | | | | | | |
| 3 | randomised trials | serious^1^ | no serious inconsistency | | no serious indirectness | | serious^2^ | | none | 16/164  (9.8%) | 49/156  (31.4%) | RR 0.3 (0.18 to 0.5) | 220 fewer per 1000  (from 157 fewer to 258 fewer) | ⊕⊕OO LOW | IMPORTANT |
|  |  |  |  |  |  |  |  |  |  |  | 31.3% |  | 219 fewer per 1000  (from 156 fewer to 257 fewer) |  |  |

^1^ Studies were of poor quality, such as not reporting in detail the specific method of random sequence generation and allocation concealment, not using blinding, incomplete reporting of patients and outcome events, or selective reporting of results.
^2^ High heterogeneity, such as point estimates that varied widely across studies, narrow or non-overlapping confidence intervals, small p-values for the test of heterogeneity, or a large I2 for the test of heterogeneity.
^3^ Confidence intervals that spanned the equilibrium line or sample sizes that did not exceed the OIS.
